# Deep Phenotyping of Obesity: Electronic Health Record–Based Temporal Modeling Study

**DOI:** 10.2196/70140

**Published:** 2025-08-20

**Authors:** Xiaoyang Ruan, Shuyu Lu, Liwei Wang, Andrew Wen, Sameer Murali, Hongfang Liu

**Affiliations:** 1 Department of Health Data Science and AI, McWilliams School of Biomedical Informatics, The University of Texas Health Science Center at Houston, 7000 Fannin St, Houston, TX, 77030, United States, 1 713-500-3924; 2 Department of Clinical and Health Informatics, McWilliams School of Biomedical Informatics, The University of Texas Health Science Center at Houston, Houston, TX, United States; 3 Department of Surgery, McGovern Medical School, University of Texas Health Science Center at Houston, Houston, TX, United States

**Keywords:** obesity, anti-obesity medication, EHR, phenotyping, precision medicine

## Abstract

**Background:**

Obesity affects approximately 40% of adults and 15%‐20% of children and adolescents in the United States, and poses significant economic and psychosocial burdens. Currently, patient responses to any single antiobesity medication (AOM) vary significantly, making obesity deep phenotyping and associated precision medicine important targets of investigation.

**Objective:**

This study aimed to evaluate the potential of electronic health records (EHR) as a primary data source for obesity deep phenotyping. We conducted an in-depth analysis of the data elements and quality available from obesity patients prior to pharmacotherapy and applied a multimodal longitudinal deep autoencoder to investigate the feasibility, data requirements, clustering patterns, and challenges associated with EHR-based obesity deep phenotyping.

**Methods:**

We analyzed 53,688 pre-AOM periods from 32,969 patients with obesity or overweight who underwent medium- to long-term AOM treatment. A total of 92 laboratory and vital measurements, along with 79 *ICD* (*International Classification of Diseases*)-derived clinical classifications software (CCS) codes recorded within one year prior to AOM treatment, were used to train a gated recurrent unit with decay-based longitudinal autoencoder (GRU-D-AE) to generate dense embeddings for each pre-AOM record. Principal component analysis and Gaussian mixture modeling (GMM) were applied to identify clusters.

**Results:**

Our analysis identified at least 9 clusters, with 5 exhibiting distinct and explainable clinical relevance. Certain clusters show characteristics overlapping with phenotypes from traditional phenotyping strategy. Results from multiple training folds demonstrated stable clustering patterns in 2D space and reproducible clinical significance. However, challenges persist regarding the stability of missing data imputation across folds, maintaining consistency in input features, and effectively visualizing complex diseases in low-dimensional spaces.

**Conclusions:**

In this proof-of-concept study, we demonstrated longitudinal EHR as a valuable resource for deep phenotyping the pre-AOM period at per patient visit level. Our analysis revealed the presence of clusters with distinct clinical significance, which could have implications in AOM treatment options. Further research using larger, independent cohorts is necessary to validate the reproducibility and clinical relevance of these clusters, uncover more detailed substructures and corresponding AOM treatment responses.

## Introduction

Obesity affects approximately 40% of adults and 15%‐20% of children and adolescents in the United States [1,2]. It is projected that 49% of adults will have obesity, and 24.2% have severe obesity by 2030 in the USA [3]. Obesity increases the risk of a wide spectrum of chronic diseases and causes profound economic and psychosocial burden. Obesity arises from a complex interplay of genetic [4-7], nongenetic [8,9], and epigenetic factors [10-13]. Due to this multifaceted nature, no single therapy, either noninvasive (eg, antiobesity medications (AOMs), dietary control, hydrogel) or invasive (eg, bariatric surgeries), can robustly predict patient response. For example, meta-analysis indicates wide interindividual response after various bariatric surgeries during longer-term follow-up [14,15]. Roux-en-Y gastric bypass of 6000 patients showed 23.1% as nonresponders 5 years after surgery [16]. Follow-up of 652 patients with sleeve gastrectomy for 7 years indicated 27.8% weight recidivism [17]. For AOMs, even the most effective glucagon-like peptide-1 (GLP-1) drug Tirzepatide claimed 10% weight loss in only 69% participants after 1 year at a relatively safe dosage [18]. Other AOMs [19] and treatment strategies like hydrogels [20] and vagal nerve blockade [21,22] generally show broader interindividual variations, which make them often an auxiliary part of a weight management plan [23-25].

The inconsistent therapeutic response makes obesity phenotyping (ie, classify obesity into subtypes) and associated precision management important targets of investigation. Earlier advancements in obesity staging [26] and phenotyping-guided pragmatic trials [27] have moved beyond the oversimplified classification by BMI [28] and demonstrated clinical values [29-31]. However, health care practitioners (HCPs) still found major obstacles in adopting them for obesity precision medicine [32]. One obstacle lies in the complexity of these strategies. For example, the trial by [27] involves tedious (eg, satiation measured by ad libitum buffet meal) and subjective (eg, hunger measured by visual analog scale) measurement processes. There are also considerable gaps between the desired and actual level of acceptance of obesity treatment guidelines by HCPs [33,34]. Another major obstacle is the lack of granularity of the phenotypic information to personalized context [32]. Phenotype-based pharmacotherapy has been successful only in rare monogenic obesity [35]. To date, the sole predictor of the long-term response of pharmacotherapy is the short-term weight loss result, which is useless for guiding the initial treatment selection [36].

Recent review articles [37,38] concluded with the necessity of obesity deep phenotyping for precision medicine, as “when considering obesity, every person should be assessed based on their own specific and unique circumstances” [32]. Echoing this statement, our recent study covering more than 1 million obesity and overweight patients indicates wide interindividual responses to any single AOM regardless of exposure lengths [39]. To date, there is a significant dearth of research on important questions like in what clinical context (eg, at the time of diagnosis; before initiating pharmacotherapy; postsurgical weight management) should we discuss “deep phenotyping,” the type or source or availability or quality of relevant data elements, the computational methodologies, and how “deep” should and can we go. To take a peek into this intricate and multifaceted research question, here we narrow down the scope to deep phenotyping before initiating pharmacotherapy, a period with direct relation to drug response and thus potential clinical impact. We propose that electronic health records (EHRs) are a valuable data source for this purpose, as they provide detailed information across patient journeys and have inspired relevant research [40,41]. Using real-world EHR data from 444,219 patients with obesity or overweight diagnosed between 2005 and 2023, we analyzed commonly available data elements and their quality before pharmacotherapy. We also tested a multimodal longitudinal deep autoencoder to examine the feasibility, data requirements, clustering patterns, and challenges of EHR-based obesity deep phenotyping.

## Methods

### EHR Database and the Case Cohort

The study is based on EHR data from the outpatient practice of the University of Texas Health Sciences Center at Houston’s McGovern Medical School. The data is transformed (from Allscripts Touchworks EHR pre-2021, GE Centricity EHR pre-2021 for billing, EPIC EHR post-2021) to Observational Medical Outcomes Partnership (OMOP) common data model (CDM) on a nightly basis to harmonize data query format. Currently, the OMOP CDM instance covers 6.5 million patients, among which approximately 3.7 million patients were documented between January 1, 2005, and December 31, 2023. From these patients, we identified 456,890 patients with either obesity (n=217,040; Textbox 1) or overweight (n=239,850) diagnosis. For the purpose of the study, overweight was defined as patients with certain comorbidities (Textbox 2) within 1 year prior to a BMI measurement ≥27, to reflect a pragmatic, risk-based approach increasingly used in personalized obesity care. From the 456,890 patients we defined the case cohort as the 32,969 patients with medium (>112 d) to long exposure to AOMs and who have no bariatric surgeries. Obesity diagnosis, comorbidities, and demographic information—such as age, race, and gender—were extracted from EHR data collected during routine clinical practice.

Textbox 1. **Concept codes for obesity diagnoses**

- Obesity: 433,736;
- Morbid obesity: 434,005;
- Localized adiposity: 438,731;
- Extreme obesity with alveolar hypoventilation: 4,100,857;
- Drug-induced obesity: 4,097,996;
- Simple obesity: 4,217,557.

Textbox 2. **Comorbidities for determining overweight**

Prediabetes, diabetes, hypertension, metabolic syndrome, obstructive sleep apnea, polycystic ovary syndrome, insulin resistance, hyperlipidemia, fatty liver, non-alcoholic steatohepatitis, coronary artery disease, cerebrovascular accident, stroke, peripheral vascular disease, congestive heart failure, colon or breast or renal or endometrial or liver cancer, osteoarthritis, or with a glycated hemoglobin level of ≥5.7 and <6.5.

### Food and Drug Administration–Approved AOMs (F-AOMs) and Off-Label AOMs (O-AOMs)

For Food and Drug Administration–Approved AOMs (F-AOMs), we considered bupropion, naltrexone, orlistat, phentermine, phentermine-topiramate, liraglutide, semaglutide, and tirzepatide that were approved by Food and Drug Administration (FDA) for obesity treatment. For Off-Label AOMs (O-AOMs), we considered bupropion, canagliflozin, dapagliflozin with or without saxagliptin, empagliflozin with or without linagliptin, lisdexamfetamine, metformin with or without (liflozin, liptin, litazone, and statin), topiramate, and zonisamide. Specifically, exposure to an O-AOM ingredient for less than 30 days without a neighboring (30 d before or after) record was removed to allow for occasional use of O-AOMs for other purposes. While most of the O-AOMs are for diabetes treatment, we make no explicit distinction between the treatment purposes when the treatment course is longer than 30 days, in which case an impact on body weight should be anticipated. For each drug ingredient, we searched the RxNav [42] database for all aliases. We then searched the Observational Health Data Sciences and Informatics Athena [43] database for concept ids of the original ingredient and all aliases. The concept IDs were combined to represent a single corresponding ingredient.

### Definition of AOM Treatment Session

An AOM treatment session represents a continuous period of exposure to generally the same active ingredients. Specifically, compared to an existing exposure record *REC_A_*, a new exposure record *REC_B_* belongs to the same treatment session (as RECA) if it (1) has exactly the same ingredient(s) as RECA and there is ≤40 days gap between start of RECB and end of RECA, or (2) has less ingredient than RECA, and has both exposure length and gap to RECA ≤40 days, or (3) has new ingredient(s) added within 40 days of initiating RECA and has exposure length ≤40 days. For all other circumstances, we consider RECB as a new treatment session. For the 32,969 patients in the case cohort, we identified a total of 53,688 AOM treatment sessions.

### Pre-AOM Period, and Data Points Sampling Scheme

We defined “pre-AOM period” as 1 year (ie, 365 d) before starting each AOM treatment session. Thus, there are 53,688 pre-AOM periods corresponding to each of the 53,688 AOM treatment sessions. For each pre-AOM period, we sampled data points every 30 days with a maximum look-back period of 30 days, resulting in 13 sampling points and 12 intervals for each feature. Notably, the interval closest to the treatment session start date spans from 35 days to 5 days before initiation. Throughout the rest of the text, we refer to the date 4 days before AOM initiation as the index date. We designed a 4-day lead time to allow for any final data collection or assessments that can be completed without interfering with the treatment process.

### Normal BMI Control Period

We randomly selected 10,000 case pre-AOM periods to match with corresponding normal BMI control periods, of patients with no obesity or overweight diagnosis and no BMI>25 kg/m². The matching process followed these criteria: (1) the case and control periods were matched by gender, (2) the age at the normal BMI measurement was within one year of the case’s age at AOM initiation, and (3) the normal BMI measurement date was within one year of the case’s AOM initiation date. In the end, 8773 case pre-AOM periods were successfully matched one-to-one with normal BMI control periods that belonged to 8185 normal-BMI patients.

### Explored Features

#### Measurements

We examined all numerical measurements recorded in the OMOP “measurement” table, which includes all lab and vital records (referred to as “measurements” in the remaining text) from the original EHR databases. The units of each measurement were harmonized to the primary unit type through manual inspection. Specifically, data quality of each pre-AOM period was defined as the average proportion of observed measurements of the 13 sampling points before AOM initiation.

#### Diagnosis Code

All *ICD-9* (*International Classification of Diseases, Ninth Revision*) diagnosis codes were cast into 265 categories of clinical classifications software (CCS) codes [44]. All *ICD-10* (*International Statistical Classification of Diseases, Tenth Revision*) diagnosis codes were mapped to *ICD-9* code through general equivalence mapping provided by Center for Medicare & Medicaid Services. CCS is a tool for clustering patient *ICD-9* diagnoses and procedures into a manageable number of clinically meaningful categories for easy presentation and statistical analysis. The CCS codes were one-hot encoded before presenting to the autoencoder model.

### Embedding of Longitudinal EHR Data

We re-engineered the GRU-D [45] based longitudinal deep learning architecture to function as an autoencoder (GRU-D-AE) for embedding the longitudinal EHR data from pre-AOM period. The basic architecture of the GRU-D model was systematically described by [45], here we recapitulate the equations for handling missing values.


$${\hat{x}}_{t}^{d}=m_{t}^{d}x_{t}^{d}+(1-m_{t}^{d})\left(\gamma_{x_{t}}^{d}x_{t^{\prime }}^{d}+(1-\gamma_{x_{t}}^{d}){\tilde{x}}^{d}\right)$$

where $m_{t}^{d}$ is the missing value indicator for feature d at timestep $t,m_{t}^{d}$ takes value 1 when $x_{t}^{d}$ is observed, or 0 otherwise, in which case the function resorts to weighted sum of the last observed value $x_{t^{\prime }}^{d}$ and empirical mean ${\tilde{x}}^{d}$ calculated from the training data for the dth feature. Furthermore, the weighting factor $\gamma_{x_{t}}^{d}$ is determined by


$$\gamma_{t}=\exp\left\{-\max(0,W_{\gamma }\delta_{t}+b_{\gamma })\right\}$$

where $W_{\gamma }$ is a trainable weights matrix and $\delta_{t}$ is the time interval from the last observation to the current timestep. When $\delta_{t}$ is large (ie, the last observation is far away from current timestep), $\gamma_{t}$ is small, resulting in smaller weights on the last observed value $x_{t^{\prime }}^{d}$, and higher weights on the empirical mean ${\tilde{x}}^{d}$ (ie, decay to mean).

**Figure 1.**
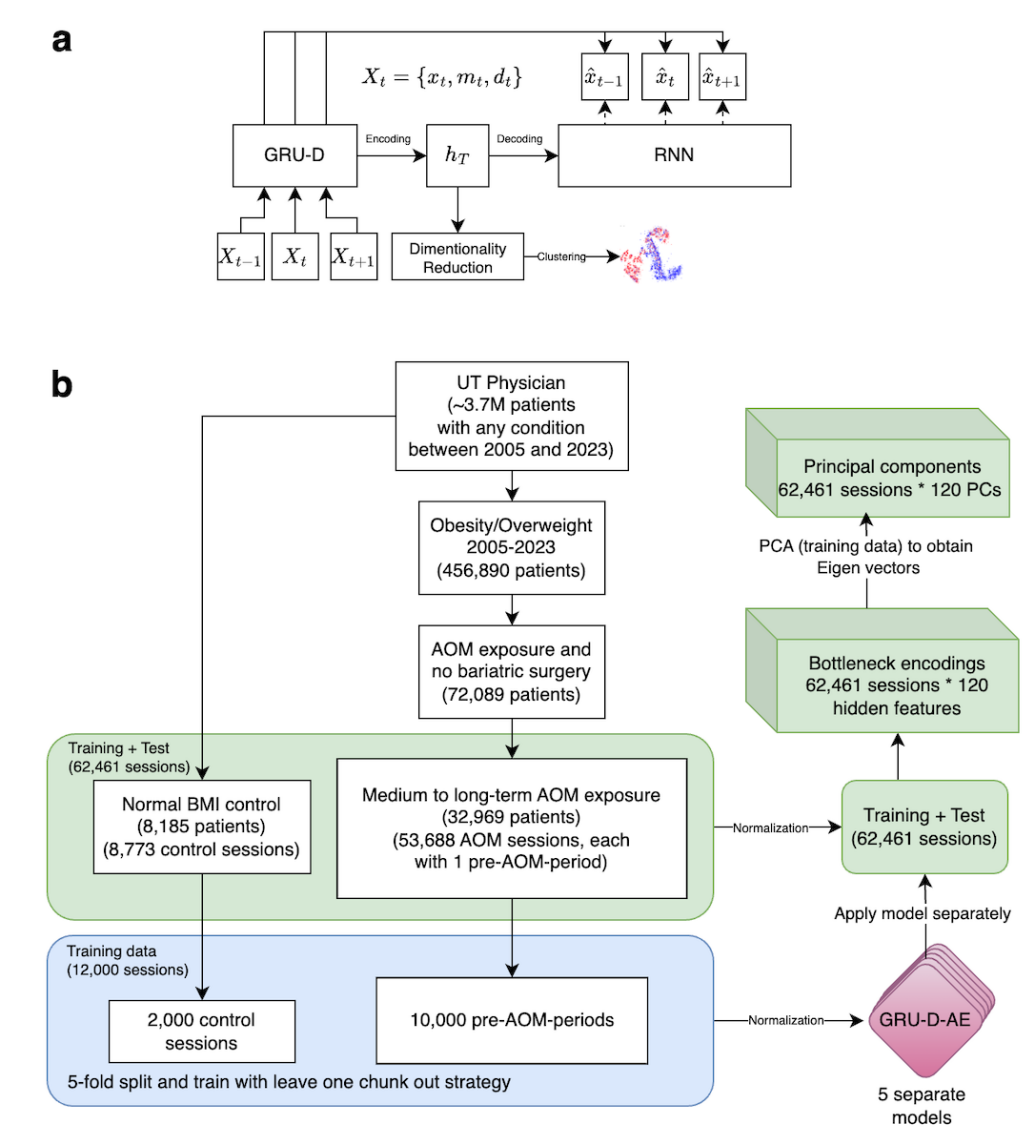
(A) GRU-D Autoencoder (GRU-D-AE) architecture. *x_t_* is the normalized feature value *m_t_* is the missing indicator (0 for missing and 1 for presence), and *d_t_* is the time since the last actual observation. The bottleneck layer *h_T_* contains the dense embedding vector used for clustering. (B) Sample processing flow and the generation of Principal Components (PCs) from longitudinal electronic health record. AOM: antiobesity medication; RNN: recurrent neural network; UT: University of Texas.

The architecture of GRU-D-AE is illustrated in Figure 1A, where *X_t_* represents all input data at timestep *t*, *x_t_* is the normalized feature value (eg, converting lab measurements to corresponding z-scores; scaling age to a 0‐1 range), *m_t_* is the missing indicator (0 for missing and 1 for presence), and *d_t_* is the time since the last actual observation. The bottleneck layer *h_T_* contains the dense embedding vector as the output of GRU-D from the last time step *T. h_T_* is then passed to a native RNN network (in this case, a native GRU model) to generate the reconstructed feature value $\underset{\_}{x}$. Figure 1B shows the data processing flow, from raw EHR data to principal components, and how GRU-D-AE functions in the process.

Specifically, the loss function for GRU-D-AE is expressed as


$$Loss=\sum_{i=1}^{N}\left(\sum_{t=1}^{T}{\left({\hat{x}}_{it}-{\bar{x}}_{it}\right)}^{2}\right)$$

Where *N* represents the number of training samples, *T* the longitudinal time steps during pre-AOM period, $\underline{x}$ the reconstructed feature value for sample *i* at time step *t*, $\hat{x}$ the original observation or, if missing, an imputed value through the GRU-D missing parameterization mechanism.

### Embedding of Static-Transformed Longitudinal EHR Data

As a baseline comparison, we conducted experiments using an atemporal sparse autoencoder (SAE) to embed static-transformed longitudinal EHR data. The static transformation involved extracting the last observed feature within the 1-year period preceding AOM initiation. Features with no observations during this period were imputed with 0 and excluded from the loss function through masking. The experiment pipeline, including the training sample indices, was identical to that used in the GRU-D-AE model (Figure 1B).

### Principal Component Analysis and Clustering Algorithm

Principal component analysis (PCA) was conducted on the embeddings generated from the training data to derive eigenvectors. These eigenvectors were then applied to the embeddings from both the training and test datasets to calculate the corresponding principal components (PCs; Figure 1B). We used the Gaussian mixture model (GMM) to perform probability-based clustering on the top 40 PCs of each embedding. To visualize the embeddings in two-dimensional space, we explored 2 methods: a scatterplot of the top two PCs and a t-SNE plot of the top 40 PCs. Cluster characteristics—including CCS prevalence rates, *z* score–transformed mean measurement levels, and missing data proportions—were visualized using a 2-way heatmap with Euclidean distance applied both rows and columns wise.

### Computational Environment

The computational environment for data analysis consisted of a Unix-based high-performance computing system with 192 CPUs. Deep learning models were developed using PyTorch version 2.3.1, implemented in Python version 3.12.2. Statistical analyses were conducted using R version 4.3.3. PCA was performed with the built-in *stats* package in R, while GMM was implemented using the *mclust* package (version 6.1.1). Two-way heatmap was implemented with the R pheatmap package (version 1.0.12). t-SNE dimensionality reduction was carried out with the *Rtsne* package (version 0.17). Data visualization tasks were completed using the ggplot2 package (version 3.5.0) in R.

### Ethical Considerations

This study was approved by The University of Texas institutional review board (IRB), and the Ethics Committee waived the need for written informed consent from participants.

The research involved secondary analysis of previously collected EHR data and was determined to be exempt from full IRB review, as it involved de-identified data with minimal risk to participants.

Informed consent was obtained from all individuals at the time of original data collection. The IRB determined that additional informed consent was not required for this secondary analysis, as the study used de-identified data and was consistent with the scope of the original consent.

All study data were de-identified prior to analysis in accordance with institutional and federal privacy regulations. No direct identifiers were used or linked, and all analyses were conducted in secure, access-controlled environments to ensure confidentiality.

No compensation was provided to individuals as this was a secondary analysis of existing data, and no direct interaction with participants occurred.

No images or other media containing identifiable information about individuals were included in the manuscript or supplementary materials. If any potentially identifiable images are included in future submissions, appropriate consent forms will be obtained and uploaded.

## Results

### Baseline Characteristics of the Study Population

From January 1, 2005, to December 31, 2023, we identified 444,309 (12%) patients with either obesity (n=204,688) or overweight (n=239,621) diagnosis. Among these patients, 72,089 (16%) were exposed to AOM therapy, with a total of 136,728 AOM treatment sessions (ie, averagely ~2 treatment sessions for each patient). 53,688 (39%) of the AOM sessions from 32,969 patients belong to medium or long-term exposure (>=112 d). The baseline characteristics of the study population were shown in Table 1. The sample processing flow is illustrated in Figure 1B.

**Table 1.** Demographics of the study population.

	Total cohort	With AOMs^a^ exposure	With medium/long term AOMs exposure
Patient count	444,219	72,089	32,969
AOM session count	—^b^	136,728	53,688
Gender			
Male	178,097 (40)	26,891 (37)	11,924 (36)
Female	266,057 (60)	45,188 (63)	21,037 (64)
Race			
White	150,802 (34)	30,425 (42)	12,752 (39)
Black or African American	108,170 (24)	17,501 (24)	8815 (27)
Asian	9499 (2.1)	1896 (2.6)	1089 (3.3)
Others	1758 (0.4)	279 (0.4)	133 (0.4)
Unknown	173,990 (39)	21,988 (31)	10,180 (31)
Age at first diagnosis	51 (37, 63)	56 (42, 66)	51 (39, 64)
Diagnosis			
Obesity	139,880 (31)	20,537 (28)	11,842 (36)
Morbid obesity	61,495 (14)	12,399 (17)	5989 (18)
Localized adiposity	1738 (0.4)	288 (0.4)	155 (0.5)
Extreme obesity	1032 (0.2)	136 (0.2)	63 (0.2)
Drug-induced obesity	456 (0.1)	141 (0.2)	83 (0.3)
Simple obesity	17 (0.004)	7 (0.01)	6 (0.1)
Overweight	239,600 (54)	38,581 (54)	14,831 (45%)

a AOM: antiobesity medication.

b Not applicable.

### Overall Feature Availability During Pre-AOM Period

During the pre-AOM period, which has potentially the most relevant data for deep phenotyping, we identified 92 measurements with ≥5% overall presence rate and nonzero standard deviation (Table S1 in Multimedia Appendix 1). The measurements with ≥70% presence rate are body weight, body surface area, BMI, height, blood pressure (BP), and heart rate. Whereas lab measurements for basic metabolic panels (eg, glucose, calcium, sodium), and lipid levels (eg, high-density-lipoprotein cholesterol, low-density-lipoprotein cholesterol, triglyceride) generally have around or ≥50% presence rate. Examining the distribution of overall feature presence rates among patient subgroups reveals no differences between males and females, nor across racial groups (ie, White, Black or African American, Asian, etc).

Out of 285 CCS categories, we identified 79 with ≥5% overall presence rate (Table S2 in Multimedia Appendix 2). The most common CCS categories are diabetes mellitus without complications (63%), hypertension (55%), hypertension with complications (55%), and hyperlipidemia (51%). Nutritional disorders, including any type of obesity diagnosis, were present in 40% of cases within one year prior to the AOM session, generally aligning with the percentage (46%) of obesity patients in our case cohort (ie, obesity + overweight).

### Temporal Windows Based Feature Presence Rate

**Figure 2.**
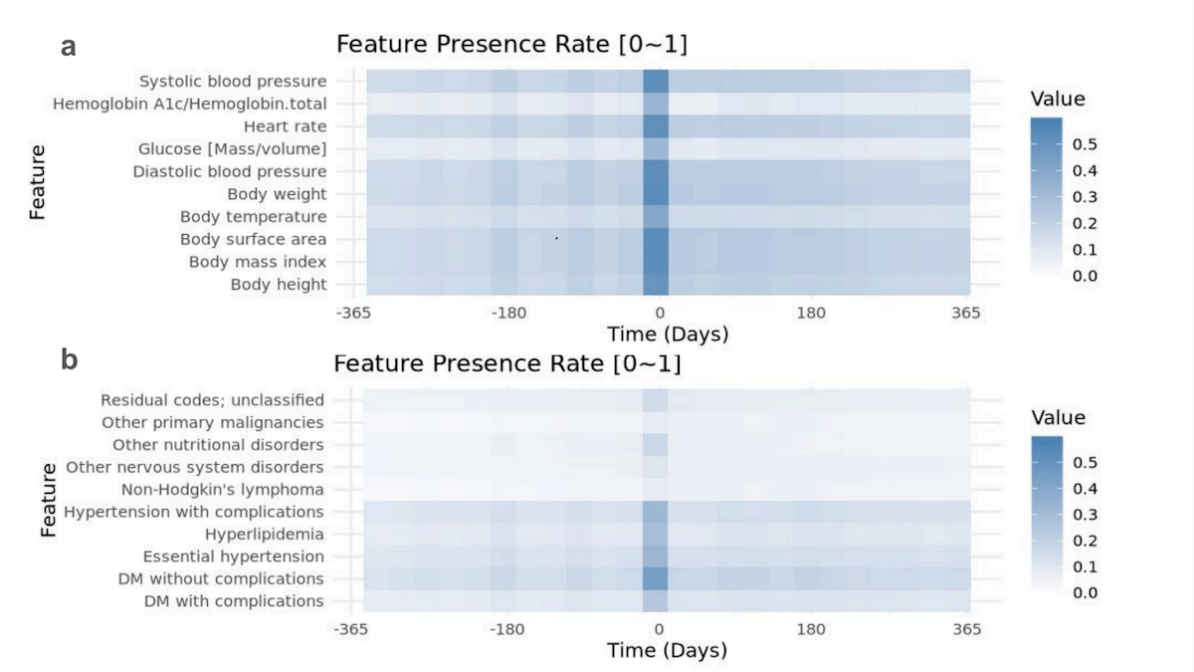
Feature presence rate of the top 10, (A) Measurements, (B) Clinical Classification Software (CCS) codes appeared within 1 year before and after initiating medium to long term Anti-Obesity Medication (AOM) therapy.

We sampled features every 30 days within one year surrounding the treatment session initiation date to examine the feature presence rates across the 24 corresponding time intervals. For both measurements (Figure 2, Figure S1 in Multimedia Appendix 3) and CCS codes (Figure 2, Figure S2 in Multimedia Appendix 3), the feature presence rates were remarkably higher in the time interval immediately before the index date (ie, Day −35 to −5), and slightly elevated around 180, 90, and 30 days before the index date. This pattern of increased feature presence was not evident after the index date and was not seen in the normal BMI control periods (data not shown). For the pre-AOM periods, 50% measurements were contributed by 22% treatment sessions. On the other hand, 9.9% patients and 11% treatment sessions had no single measurement during this period.

### Feature Presence Rate Throughout Years

Analyzing feature presence rates from 2005 to 2023 revealed a general upward trend for both measurements and CCS codes over the years. Specifically, the Mann-Kendall nonparametric trend test showed that all 92 measurements have significantly increased presence rates (*P*≤.05) over time. The top five measurements with the highest increase are microalbumin, creatinine in urine, high-density-lipoprotein cholesterol, total cholesterol, and ferritin in serum or plasma. Additionally, 50 out of the 79 CCS categories demonstrated a significant increase in presence rates over the years, with the top five being anxiety disorders, nutritional deficiencies, other acquired deformities, other non-traumatic joint disorders, and screening and history of mental health and substance abuse codes (Figure S3 in Multimedia Appendix 3). Two CCS categories—chest pain and skin and subcutaneous tissue infections—showed a significant (*P*≤.05) or marginally significant (*P*≤.10) decrease in presence rates over the years.

### Encoding and Reconstruction of Longitudinal EHR Data

We experimented with various bottleneck layer neuron sizes (n=40, 60, and 120) for the GRU-D-AE model. Generally, larger bottleneck sizes allow the autoencoder to capture more intricate temporal structures. Figure S4 in Multimedia Appendix 3 shows the GRU-D-AE based reconstructions of 171 temporal features (92 measurements and 79 CCS codes) from a single patient, using 5-fold cross-validation with a bottleneck layer of 120 neurons. Comparing results across the 5 models indicates slightly different imputation patterns across temporal steps and successful reconstruction of input feature patterns at high levels. For the remainder of the paper, we present results exclusively based on 120 bottleneck layer size, as this configuration demonstrated optimal performance.

### Clustering of Case Pre-AOM Periods

**Figure 3.**
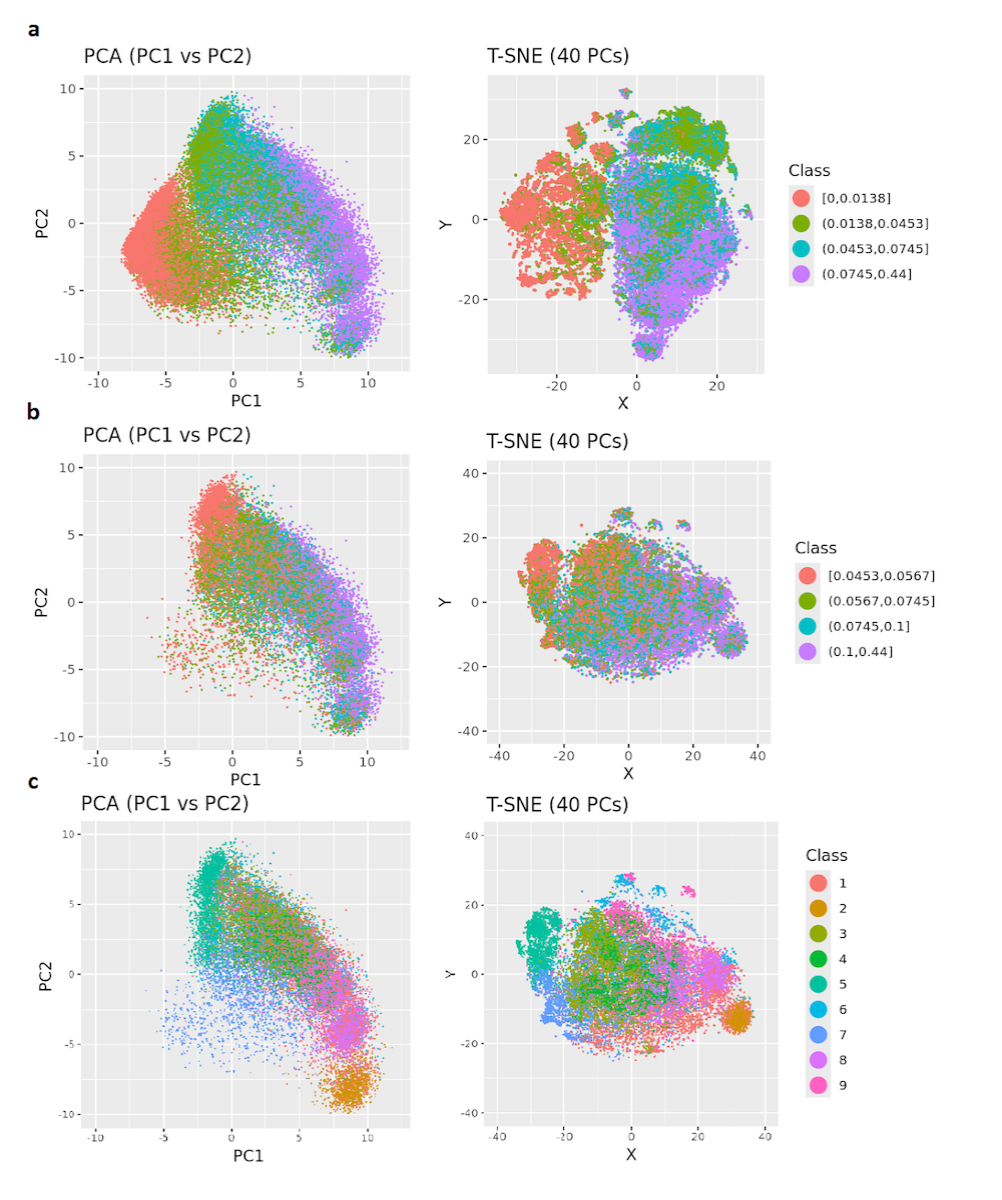
GRU-D autoencoder (GRU-D-AE) based clustering of case pre-Anti-Obesity Medication (pre-AOM) periods. (A) Coloring all pre-AOM periods according to data quality quartiles. (B) Low-quality data points (below the median) are removed, with the remaining points colored by data quality quartiles. (C) Colored by Gaussian Mixture Model (GMM)-based clustering of the high-quality data points (Principal component analysis (PCA) plot was dimmed to highlight cluster center).

By encoding the pre-AOM periods and projecting them onto principal component (PC) spaces, we visualized clustering patterns in lower-dimensional spaces, as shown in Figure 3. Specifically, Figure 3A highlights the distribution of pre-AOM periods across different data quality quartiles. Data points from the bottom quartile (red) are densely packed in the lower-left corner, while those from the top quartile (purple) are more dispersed and form distinct clusters. The T-SNE plot with top 40 PCs (explained 80% variance) reveals a complete separation of bottom quartile data points and partial separation of 2nd quartile data points. Importantly, applying the same pipeline (Figure 1B) to each of the 5-fold models shows highly reproducible clustering patterns, with different projection angles (Figure S5 in Multimedia Appendix 3). After removing low-quality pre-AOM periods (ie, the 1st and 2nd quartiles), the remaining high-quality periods (ie, the 3rd and 4th quartiles) form clusters that are less relevant to data quality (Figure 3B). GMM clustering of the 40 PCs of the high-quality periods suggests at least 9 distinct clusters within the case group (Figure 3C). Notably, GMM-based clusters may align (eg, clusters 2, 5, 7) or differ (eg, clusters 1, 6, 9) from the visually identifiable cluster centers based on the two PCs (Figure S6 in Multimedia Appendix 3). The visual separation between clusters improves slightly with T-SNE-based dimensionality reduction (Figure 7 in Multimedia Appendix 3). When the clustering results are colored by traditional obesity diagnosis categories (eg, drug-induced obesity, extreme obesity, localized adiposity, morbid obesity), no clear relationship emerges between traditional obesity diagnoses and the EHR-based clustering of pre-AOM periods (Figure S8 in Multimedia Appendix 3).

### Clustering of Case Versus Control Periods

**Figure 4.**
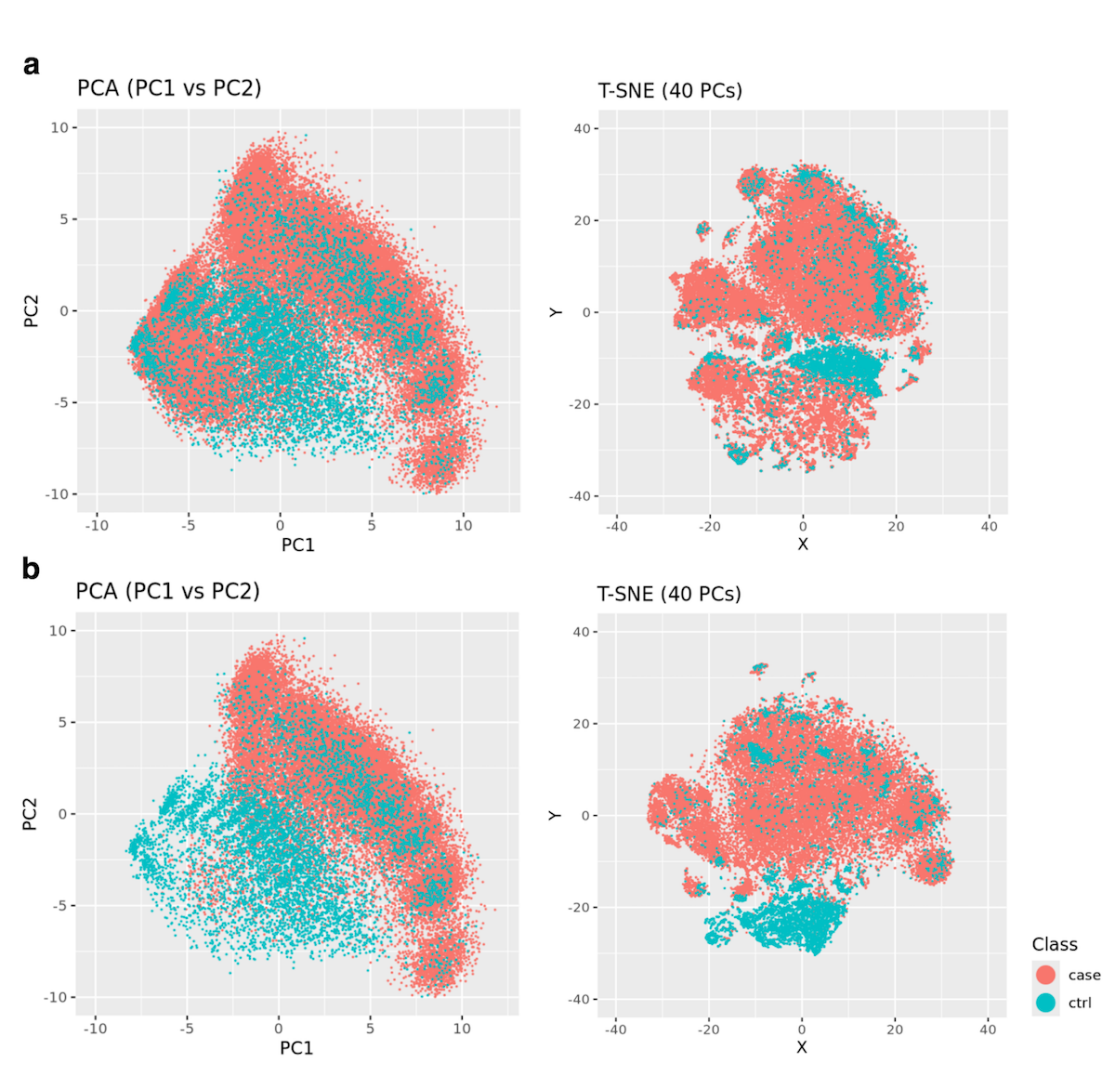
Clusters of pre-Anti-Obesity medication (pre-AOM) periods in obesity cases versus normal Body Mass Index (BMI) controls. (A) All case data points versus control data points. (B) High quality case data points versus control data points.

Visualization of the top two PCs and T-SNE plot (Figure 4) from the case pre-AOM periods and the control periods showed clear separation of the majority of control periods from the high-quality case periods. However, a minor proportion of the control periods formed clusters with centers overlapping the case group on the top two PCs (Figure 4A), with no clear pattern of overlap on the T-SNE plot (Figure 4B).

### Clustering of Multiple Pre-AOM Periods From the Same Patient

**Figure 5.**
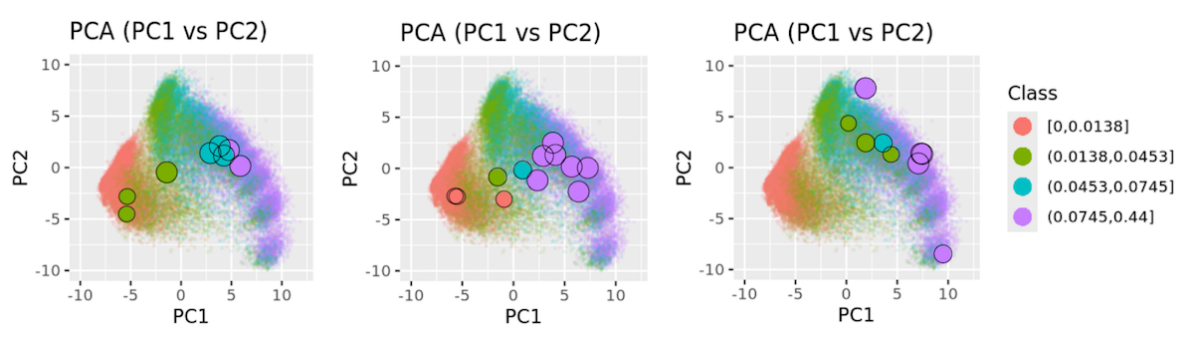
Illustration of three patients, each with multiple Anti-Obesity Medication (AOM) sessions and respective pre-Anti-Obesity Medication (pre-AOM) periods. Smaller point sizes indicate pre-AOM periods earlier in time, while colors represent different data quality quartiles.

For patients with multiple pre-AOM periods from corresponding multiple AOM treatment sessions, their clustering behavior is influenced by both data quality and intrinsic physiological factors reflected through measurements and diagnosis status. In Figure 5, all three patients exhibit improved data quality over time (later pre-AOM periods are indicated by larger dots, which generally have better data quality), with varying degrees of clustering tightness in the high-quality pre-AOM periods (represented by cyan and purple points).

### Characterizing GMM-Based Clusters of Pre-AOM Periods

**Figure 6.**
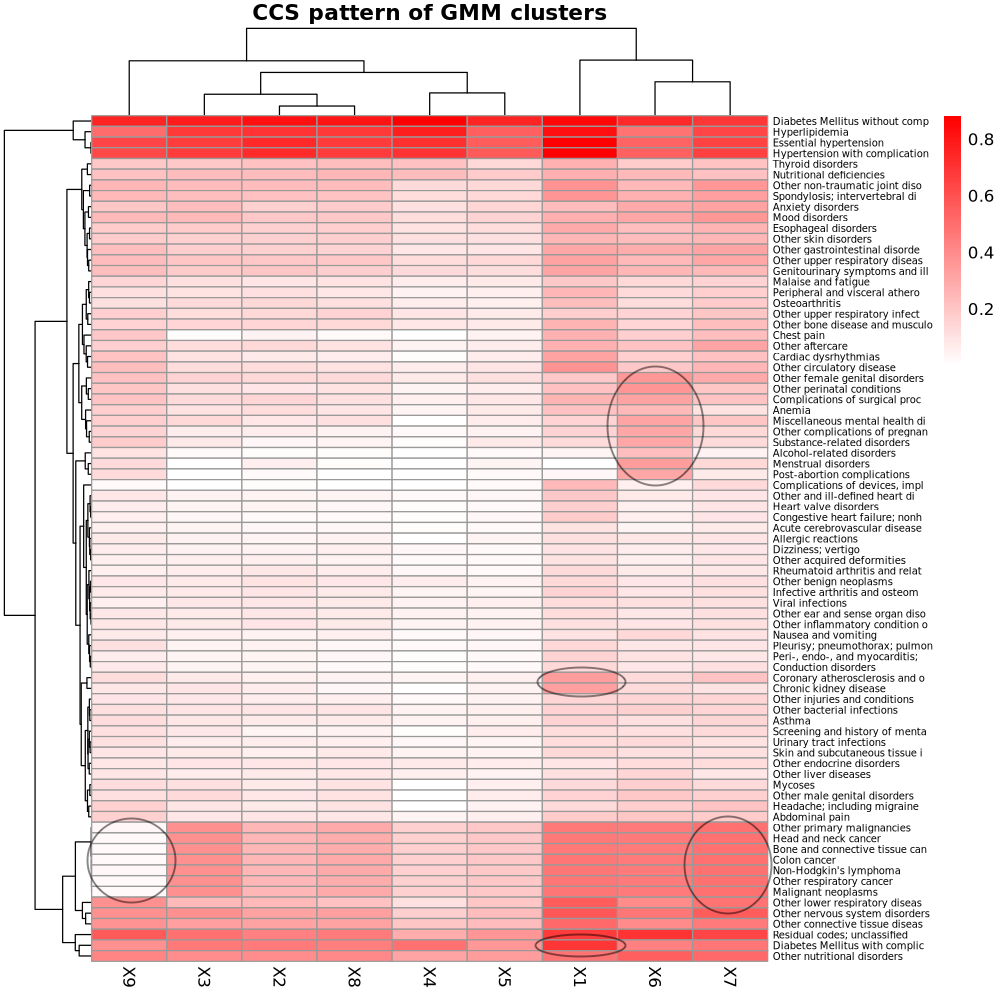
Two-way clustering of Clinical Classification Software (CCS) diagnosis prevalence rates against Gaussian mixture model (GMM)–based clusters of pre-Anti-Obesity Medication (pre-AOM) periods. Prevalence rates are calculated based on any diagnoses made within one year prior to the index date. Black circles indicate the defining characteristics of the corresponding clusters, which are referenced in the main text for guided interpretation.

**Figure 7.**
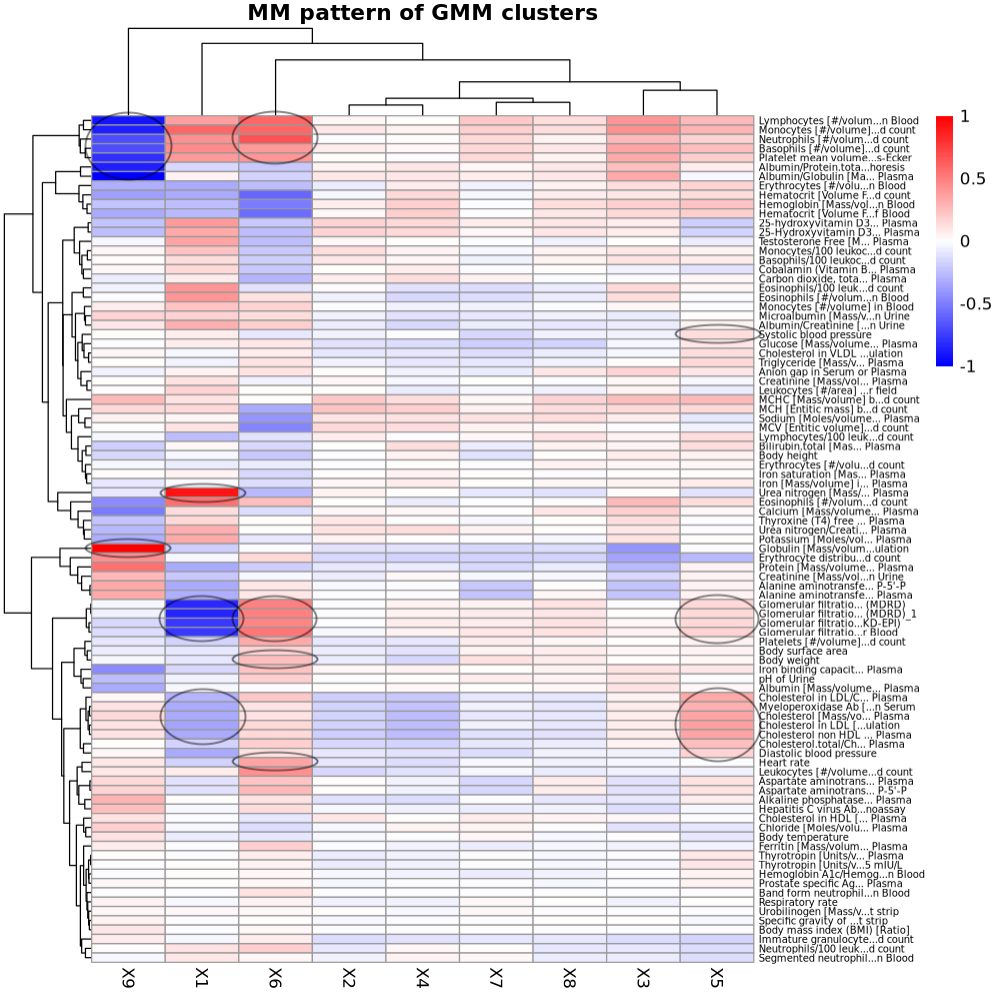
Two-way clustering of mean measurement values (z-score transformed) against Gaussian mixture model (GMM)–based clusters of pre-Anti-Obesity Medication (pre-AOM) periods. The measurement values represent the most recent observations within one year prior to the index date. Black circles indicate the defining characteristics of the corresponding clusters, which are referenced in the main text for guided interpretation.

We conducted two-way clustering of the GMM-based clusters of pre-AOM periods against CCS prevalence rates (Figure 6), average measurement values (Figure 7), and temporal measurement presence rate (Figure S9 in Multimedia Appendix 3). Here we briefly describe the 5 clusters with remarkable clinical relevance.

The *1st cluster* is distinguished by the highest prevalence of chronic kidney disease (CKD), coronary atherosclerosis, and diabetes mellitus with complications (Figure 6 X1). This cluster also shows notably low glomerular filtration rates, elevated blood urea nitrogen, and comparatively lower levels of low-density cholesterols and myeloperoxidase (Figure 7 X1).

The *5th cluster* is primarily characterized by the highest levels of low-density lipoprotein, very-low-density lipoprotein, triglyceride, systolic or diastolic blood pressure (SBP and DBP), and myeloperoxidase among all clusters. This cluster also has slightly higher glomerular filtration rates than other clusters except cluster 6 (Figure 7 X5). However, its disease prevalence rates are not significantly distinct from those of other clusters (Figure 6 X5).

The *6th cluster* is marked by a higher prevalence of reproductive and genital health disorders (eg, female genital disorders, menstrual disorders, and postabortion complications; Figure 6 X6). It also features higher heart rates, the highest body weight and glomerular filtration rates, and increased levels of leukocytes, lymphocytes, monocytes, neutrophils, and glucose levels (Figure 7 X6).

The *7th Cluster* is characterized by a high level of primary malignancies (eg, head and neck cancer, bone and connective tissue cancer, and colon cancer) (Figure 6 X7), the highest presence rate of temporal anthropometric and physiological measurements (eg, body height, body weight, body temperature, BP, heart rate), and a lower to average presence rate of other more advanced lab tests. It is also featured with a mildly higher body weight (Figure S9 X7 in Multimedia Appendix 3).

The *9th cluster* is notable for significantly lower prevalence rates of primary malignancies (Figure 6 X9), and remarkably lower leukocyte levels (including lymphocytes, monocytes, and neutrophils) alongside elevated globulin levels (Figure 7 X9).

No notable difference was observed between the clusters on average ADI-based SDOH (social determinants of health) factors including median household income, mean education percentage, and mean insurance percentage (data not shown). Inspecting demographic characteristics (ie, age, gender, race) indicates a relatively lower male proportion in cluster 6, slightly higher proportion of black people in cluster 9, and slightly older age in cluster 1 (data not shown).

Interestingly, clusters characterized by distinct clinical traits (eg, reproductive and genital health disorders in Cluster 6 and the absence of primary cancer in Cluster 9) were not visually distinguishable on the first two PCs (Figure S6 in Multimedia Appendix 3) and only formed vague boundaries in the 40-PC based T-SNE plot (Figure S7 in Multimedia Appendix 3). In contrast, clusters that showed clear separation in the low-dimensional spaces (eg, Cluster 2) may not have distinct clinical characteristics as other clusters, at least based on the available study features. We also tried GMM clustering using just the top two PCs, which produced visually distinguishable clusters but lacked meaningful clinical differentiation (data not shown).

### SAE-Based Embedding and Clustering of Pre-AOM Periods

As a baseline comparison, we evaluated the SAE-based embedding of static-transformed pre-AOM periods and summarized the results here. Using the same bottleneck layer size (n=120), the SAE model reliably reconstructed the static-transformed features (Figure S11 in Multimedia Appendix 3). However, embeddings generated by the SAE model do not exhibit visually distinct clusters on the top two PCs (Figure 12a in Multimedia Appendix 3). When colored by data quality quartiles, pre-AOM periods with lower data quality appear less separable (Figure S12a in Multimedia Appendix 3), aligning with the results observed in the GRU-D-AE model (Figure 3A). Additionally, SAE-based embeddings show weaker differentiation between pre-AOM periods across different data quality quartiles (Figure 12 in Multimedia Appendix 3). For high-quality pre-AOM periods, GMM clustering reveals less defined cluster boundaries on both the top two PCs (Figure S13a in Multimedia Appendix 3) and the T-SNE plot (Figure 13b in Multimedia Appendix 3). Overall, the SAE-based model demonstrates less distinct separation between normal BMI controls and high-quality case pre-AOM periods (Figure S14 in Multimedia Appendix 3).

## Discussion

### Background

In this preliminary work, we outlined a workflow for obtaining EHR-based fine-grained obesity phenotypes at per patient visit level prior to initiating AOM therapy. Since EHR chronicled a patient’s physiological and pathological status, this may potentially help with AOM treatment decision making. This study aligns with the growing interest in classifying obesity into distinct subtypes and the adoption of the plural term “obesities” [46,47]. It also lays the foundation for using EHR-based digital fingerprints as an alternative to traditional phenotyping approaches, which have shown potential in certain contexts [27,48] but not yet widely adopted by healthcare providers for precision obesity treatment due to their labor-intensive nature and insufficient granularity [32].

### Principal Findings

Our analysis identified at least 9 distinct clusters before AOM initiation. Five clusters show clear clinical relevance independent of traditional obesity diagnoses (eg, extreme obesity, localized adiposity). Below is a brief overview of the clinical significance of these clusters: Cluster 1 primarily includes patients with CKD and metabolic disorders. Obesity drives risk factors for CKD, most notably hypertension and type 2 diabetes, and also through obstructive sleep apnea. Hypertension is the #1 modifiable risk factor for CKD [49], while type 2 diabetes remains the leading cause of CKD worldwide [50]. Obstructive sleep apnea contributes to nocturnal hypoxia and worsens hypertension — both of which accelerate kidney injury. Beyond these indirect pathways, visceral adiposity itself promotes a proinflammatory state (marked by TNF-alpha, IL-6, MCP-1) that drives renal fibrosis and activates the renin-angiotensin-aldosterone system, leading to glomerular hyperfiltration. Additionally, increased intra-abdominal pressure from central obesity can directly alter renal hemodynamics, further contributing to hyperfiltration and progressive kidney damage. On the other hand, CKD can lead to water retention and increased body weight. The bidirectional relationship between obesity and CKD [51] makes it critical in selecting appropriate anti-obesity therapies [52]. For example, a recent review article concluded that bariatric surgery should be considered in morbidly obese adults with CKD to improve the cardio-metabolic status and kidney outcomes [53]. Cluster 5 includes patients with obesity-related hyperlipidemia, elevated atherogenic lipoproteins, and higher blood pressure, but fewer severe comorbidities and clinical measurements than other clusters—possibly reflecting better overall health, shorter obesity duration, or fewer complications. Limited healthcare access may also contribute to underreported comorbidities. These patients may have a lower genetic predisposition to visceral fat accumulation, allowing them to tolerate higher BMI without metabolic dysfunction. This underscores the limitations of BMI as a sole indicator of obesity-related morbidity and highlights the need for more precise phenotyping to capture the true burden of disease. *Cluster 6* includes individuals with reproductive and genital health disorders, often related to hormonal imbalances and insulin resistance. Unlike Cluster 5, these individuals are more likely to seek medical care due to the impact of these conditions on reproductive health [54]. Recent evidence has linked maternal diabetes to neurodevelopmental disorders like autism, ADHD, intellectual disability, and other learning disorders [55]. Given maternal obesity is a driving risk factor for gestational diabetes, this cluster represents an important demographic for prenatal intervention. For example, metformin and GLP-1 receptor agonists are potential AOM options for patients in this cluster. By enhancing insulin sensitivity, metformin not only supports weight loss but also helps regulate menstrual cycles and improve ovulation in obese women [56]. Similarly, GLP-1 receptor agonists may contribute to hormonal balance and enhance fertility outcomes [57,58]. Cluster 7 predominance of primary malignancies with low frequency of cancer-related lab tests may be representative of patients who have a history of cancer and are seeking medical weight management to reduce their risk of recurrence [59]. In patients with obesity, those who underwent bariatric surgery had a 57% lower risk of cancer-related death compared to non-surgical patients matched for pre-operative BMI [60]. Bariatric surgery recipients also have a 25% lower incidence of cancer compared to a nonsurgical comparison group matched for preoperative BMI [61]. Both of these examples demonstrate the significance of weight loss interventions on cancer risk and mortality. The remarkably lower inflammatory response of Cluster 9 may be caused by less visceral adiposity and more subcutaneous adiposity. This may be an overall healthier cluster with a lower threshold for seeking treatment. Compared to visceral adiposity, subcutaneous adiposity allows for more flexible treatment options. For instance, central abdominal adiposity—a type of subcutaneous fat—can be addressed through noninvasive or minimally invasive procedures such as cryolipolysis, high-intensity focused ultrasound, nonthermal ultrasound, radiofrequency, and injection lipolysis [62]. Prospective surveillance of cluster 9 may prove fruitful in understanding if there are early clinical features that could help identify if these individuals fractionate into new clusters or previously defined clusters. These observations highlight the method’s potential to uncover unique patient groups, marking an important first step in digital phenotyping. Future steps would be to test cluster stability in a larger cohort and incorporate additional environmental, psychological, behavioral, functional factors, and even microbiome profiles or genetic markers. Finally, linking these phenotypes to treatment responses would provide more comprehensive insights and reveal any subtle subgroup patterns.

### Comparison to Prior Work

The EHR-based clusters emphasize clinical manifestations and comorbidities, whereas traditional phenotyping, as described by [27], classifies obesity within a behavioral and metabolic framework (ie, hungry brain, emotional hunger, hungry gut, and slow burn). While there is no exact mapping between the two, certain overlaps exist. For example, *Cluster 1* shares features with both the hungry brain and hungry gut phenotypes, reflecting advanced metabolic dysfunction (eg, diabetes and coronary atherosclerosis) and CKD associated with chronic overeating. *Cluster 5* aligns with the emotional hunger phenotype, given its hyperlipidemia, high levels of atherogenic lipoproteins, elevated blood pressure, and fewer comorbidities. *Cluster 9* corresponds to the slow burn phenotype, with its low prevalence of malignancies, reduced systemic inflammation, preserved renal function, and absence of dramatic metabolic derangements, consistent with slower metabolic turnover. These potential overlaps suggest combining the strengths of each approach may provide a more complete understanding of obesity and its related disorders.

### Future Direction of GRU-D-AE for Encoding Longitudinal EHR

We demonstrate the proposed GRU-D-AE architecture, along with its error function, effectively captures the nuances of high-dimensional, extremely sparse longitudinal EHR data. We also show that embeddings from native longitudinal data provide more clearly separated and distinct clusters than embeddings from static-transformed data. These characteristics make the architecture a highly valuable candidate for encoding longitudinal EHRs of obesity, as well as various other diseases. However, there are several caveats that deserve discussion and further investigation. First, the model exhibits a certain level of arbitrariness in handling missing values in different training folds. This behavior aligns with expectations, as the error function primarily minimizes the difference between input features (both observed and imputed) and reconstructed features. As a result, the model may exploit shortcuts (eg, imputing all missing values as zero, as an extreme example) to satisfy the error function. A similar concern was raised by Daniel Jarrett et al [63], who proposed target-embedding autoencoders, co-training the autoencoder with a target value to enhance stability. In another study [64], using target variables and clinical confounders improved the stability of embeddings in complex biomedical datasets. Nonetheless, selecting appropriate co-training targets (eg, clinical outcomes, BMI trajectories, treatment responses) for deep phenotyping during pre-AOM periods remains challenging and is an important area for future investigation. Second, unlike the arbitrariness in missing data imputation, the top two PCs from the five training folds consistently formed similar clustering patterns, differing only in orientation. Moreover, examination of diagnosis and measurement characteristics of clusters in other training folds revealed highly consistent clinical traits (data not shown). These findings suggest that the bottleneck layer may capture essential temporal feature characteristics independent of the missing data imputation methods. Future studies should carefully assess the impact of imputation mechanisms on cluster stability.

### Challenges

#### Visualization of Complex Disease Phenotypes

At this stage, we observed no obvious connection between visual separation in low-dimensional spaces, particularly the top two PCs, and clinical relevance. Even with advanced dimensionality reduction techniques like T-SNE, there are generally no clear-cut boundaries between clinically distinct clusters. The findings highlight the challenges of visualizing complex diseases which often cannot be adequately captured in low-dimensional representations. Further research, such as MapperPlus [65] and other open frameworks [66], will be helpful to explore how to effectively visualize these clusters in a more clinically meaningful way beyond the static heatmap employed in current study.

#### Considerations for Low-Quality Samples

In this study, we defined data quality as the observed proportion of lab or vital measurements within 1 year before AOM initiation. While this may be arbitrary given the anticipatable variation in feature availability across time and health care systems, it provides an intuitive reflection of how well a patient is documented by the EHR system during the pre-AOM period. Given the observation that pre-AOM periods with above median measurement proportion (ie, 0.0453) formed clusters generally independent of data quality levels, we propose 5% as a cut-off threshold, below which the pre-AOM periods might be less distinguishable from each other. On the other hand, further research is needed to carefully assess the source of these low-quality pre-AOM periods (eg, limited access to health care, first-time visits, and fewer comorbidities).

#### Feature Variability in High-Dimensional EHR

The laboratory and vital measurements used in this study are derived from the most commonly available features among obesity patients in the current EHR system. However, feature availability can vary over time and across healthcare systems. This variability, combined with the high dimensionality of EHR data, poses challenges for ensuring feature consistency, which can significantly affect the stability and reliability of clustering results. Bellman first introduced this issue in 1957, referring to it as the “curse of dimensionality” [67]. Similar challenges [68-71] are prevalent in the medical field, where large feature sets and limited sample sizes are common. Specifically, Loftus et al [70] reviewed these challenges in disease phenotyping, highlighting the importance of cohort representativeness and discussing caveats in data outliers, missing data, categorical variables, scaling, data transformation, and feature selection. For obesity, while certain strategies may improve cluster stability and potential clinical utility in the pre-AOM period, substantial challenges remain. Further research combining large cohorts with diverse demographic and SDOH backgrounds and intricately designed clustering approaches is needed to generate a holistic view that can inform clinical practice.

### Limitations

In this exploratory study, we only shed light on a few limited aspects of obesity deep phenotyping from the perspective of EHR. Below, we outline key limitations and suggest areas for future research. First, although Houston is one of the most ethnically and racially diverse cities in the United States, this study is still subject to the limitations inherent in a single-site study, such as selection bias, environmental and institutional influences, and regulatory differences. Broader studies conducted at the state or national level are necessary to validate the generalizability of the findings and to uncover more nuanced clustering structures. Second, due to the limited sample size, we only considered laboratory/vital measurements, diagnosis codes for deep phenotyping, which do not provide comprehensive coverage of the factors that may influence obesity phenotypes (eg, medications, social determinants of health, and genetic/epigenetic information, demographics). Future research should include these factors as phenotyping features or examine clustering patterns across different subgroups (eg, age subgroups). Third, this study focuses on clustering patterns during the pre-AOM period, but it does not explore how different clusters respond to various AOMs in body weight, BMI, lipid levels, and side effects. Given the numerous types, combinations, and exposure lengths of AOMs, this area needs to be thoroughly investigated in larger cohorts. Fourth, we did not distinguish treatment intent when the course of O-AOMs (eg, bupropion and metformin) extended beyond 30 days, based on the assumption that a sustained treatment duration is likely to affect body weight. A more refined study could exclude non–obesity-related treatment purposes by reviewing clinical notes. Finally, we demonstrate that data quality significantly influences the visual separability of the top two PCs. However, due to space limitations, we did not delve deeply into the sources of low-quality pre-AOM periods (eg, limited access to healthcare, first-time visits, fewer comorbidities) or how changes in data quality affect a given patient’s clustering behavior. Further investigation into these aspects is needed to better understand the context in which EHR-based deep phenotyping can be faithfully applied in clinical practice.

### Conclusions

Obesity is a complex, chronic condition, and its multifaceted nature poses significant challenges for deep phenotyping and precision medicine. Here we demonstrated that longitudinal EHR data can potentially serve as a valuable resource for deep phenotyping during the pre-AOM period at the individual patient visit level. Our analysis revealed the presence of clusters with distinct clinical significance, which could have an implication on AOM treatment options. Further research using larger, independent cohorts is necessary to validate the reproducibility and clinical relevance of these clusters, uncover more detailed substructures, and assess cluster-specific responses to AOM treatment.

## Supplementary material

10.2196/70140 Multimedia Appendix 1 92 measurements within 1 year before exposure to antiobesity medication.

10.2196/70140 Multimedia Appendix 2 Clinical classifications software codes within 1 year before exposure to antiobesity medication.

10.2196/70140 Multimedia Appendix 3 Additional figures.

## Abbreviations

AOM: Antiobesity Medication
BP: blood pressure
CCS: clinical classifications software
CDM: Common Data Model
CKD: chronic kidney disease
EHR: electronic health record
F-AOM: Food and Drug Administration–approved antiobesity medication
FDA: Food and Drug Administration
GMM: Gaussian mixture model
HCP: health care practitioner
*ICD-10*: *International Statistical Classification of Diseases, Tenth Revision*
*ICD-9*: *International Classification of Diseases, Ninth Revision*
IRB: institutional review board
O-AOM: Off-label AOM
OMOP: Observational Medical Outcomes Partnership
PC: principal component
PCA: principal component analysis
SAE: atemporal sparse autoencoder
SDOH: social determinants of health
