# Supplementary material for: Deep Phenotyping of Obesity: Electronic Health Record–Based Temporal Modeling Study
Source: J Med Internet Res. 2025 Aug 20;27:e70140. doi: 10.2196/70140 (PMC12373304; doi:10.2196/70140)
Supplement: Multimedia Appendix 3 [file jmir-v27-e70140-s003.docx]

**
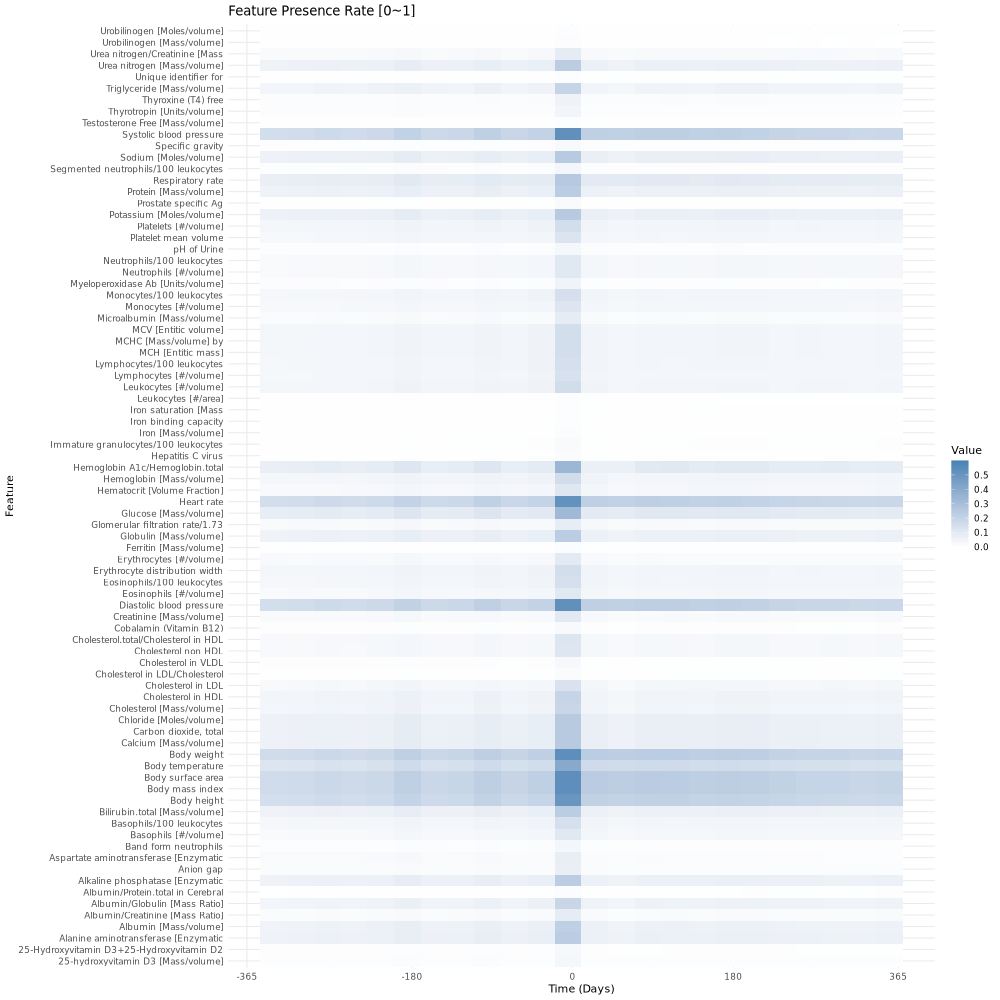
**

**Supp Figure 1** Measurement presence rate within 1 year before (i.e. pre-Anti-Obesity Medication (pre-AOM) period) and after initiating medium to long term AOM therapy

**
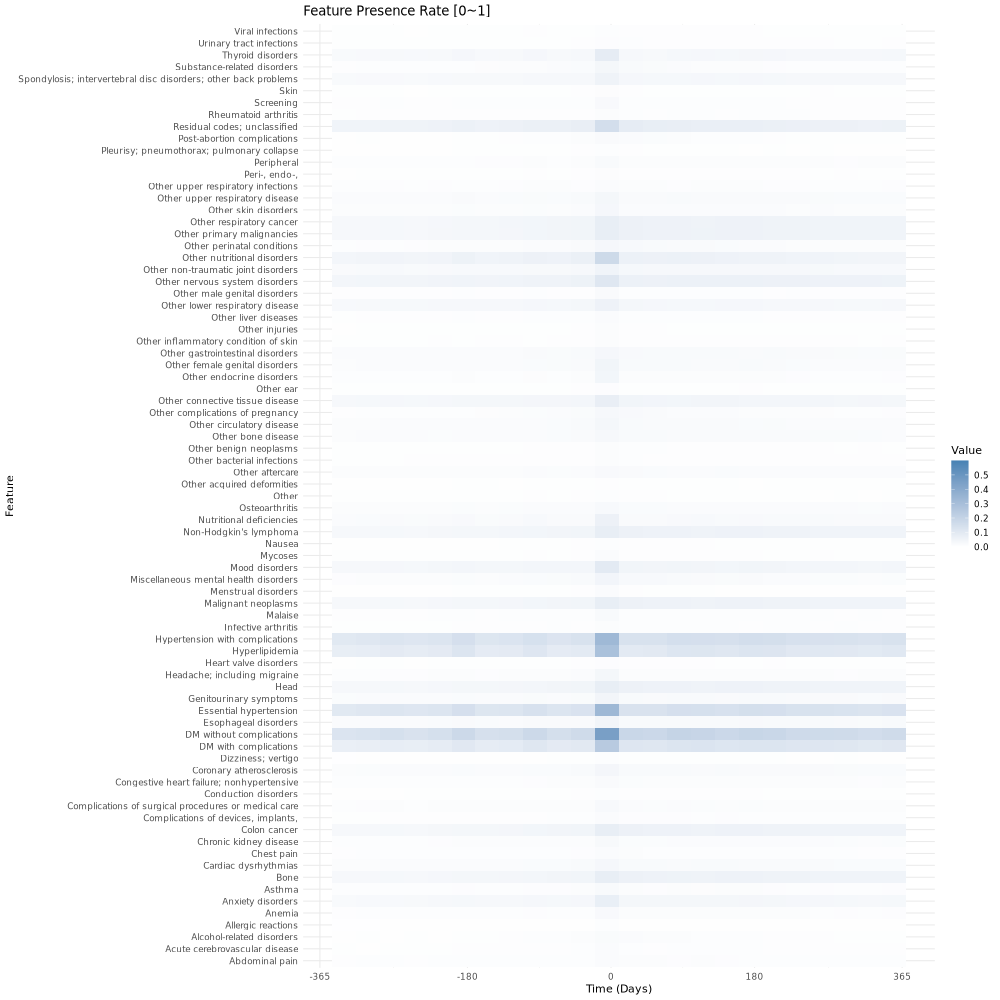
**

**Supp Figure 2** Clinical Classification Software (CCS) code presence rate within 1 year before (i.e. pre-Anti-Obesity Medication (pre-AOM)period) and after initiating medium to long term AOM therapy


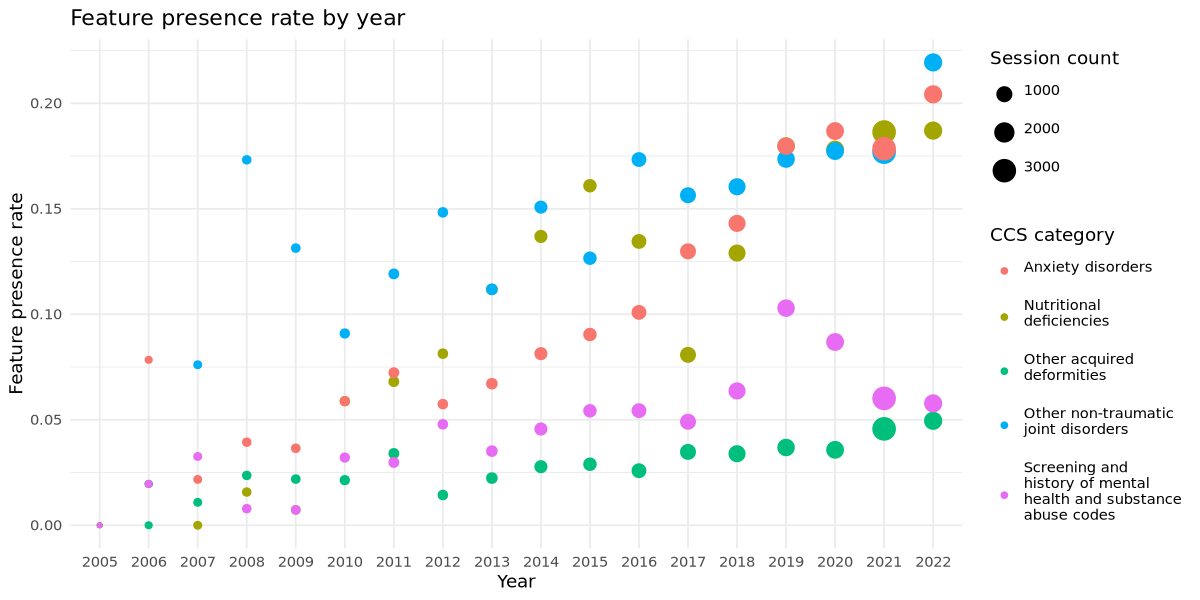


**Supp Figure 3** Top 5 Clinical Classification Software (CCS) categories with significantly increased presence rate throughout years.

**
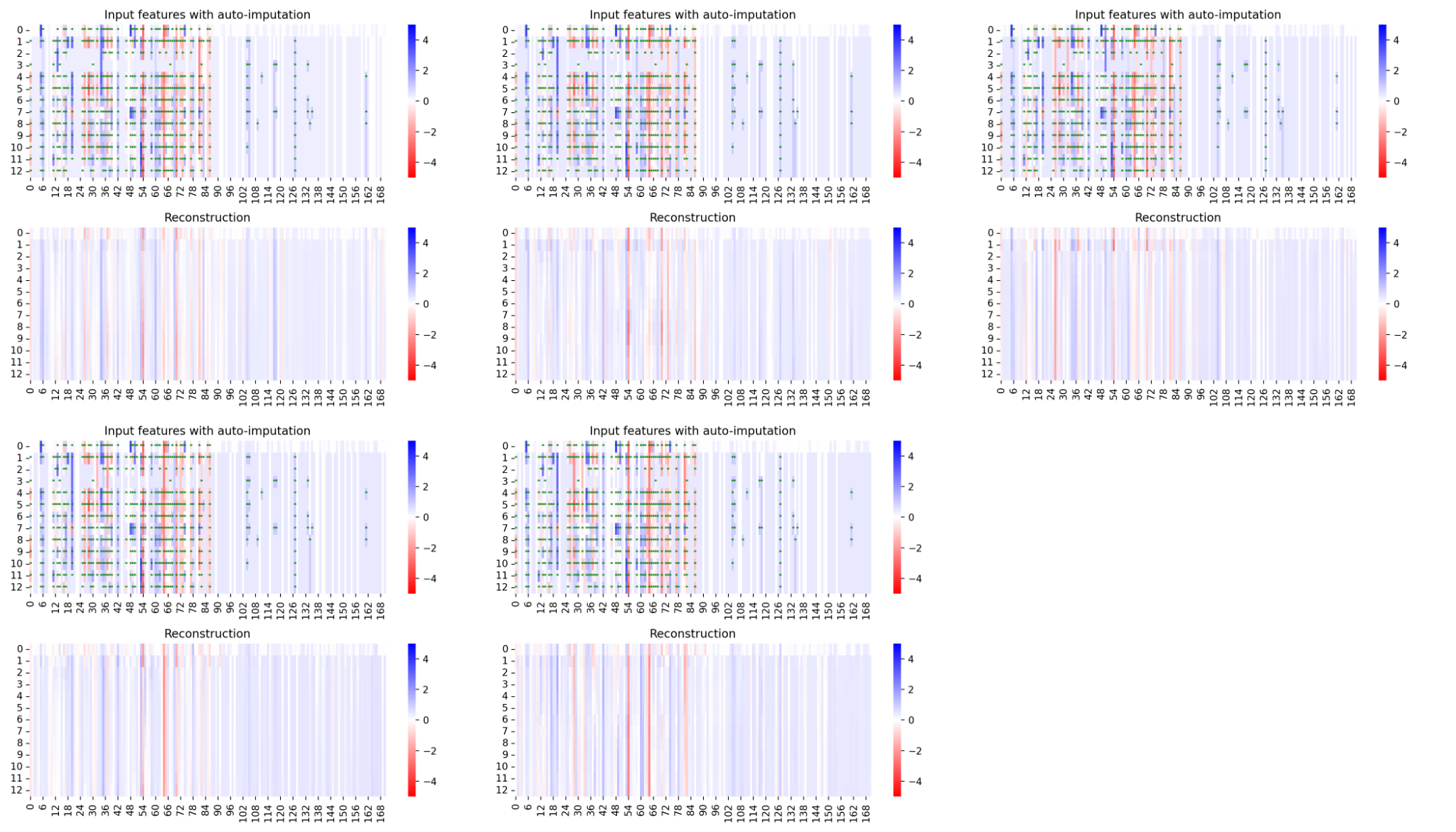
**

**Supp Figure 4** GRU-D Autoencoder (GRU-D-AE) based auto-imputation and reconstruction of a single patient's Electronic Health Record (EHR) profile during the pre-Anti-Obesity Medication (pre-AOM) period. The five subplots correspond to the outcomes of a 5-fold cross-validation using a leave-one-fold-out training approach. In each subplot, the upper section shows the original recorded values (green dots) and the imputed data for 171 longitudinal features (x-axis), each spanning 13 time points (y-axis) covering the period from 365 days to 5 days before AOM initiation. The lower section illustrates the reconstructed EHR profile after passing through a bottleneck layer consisting of 120 neurons.

**
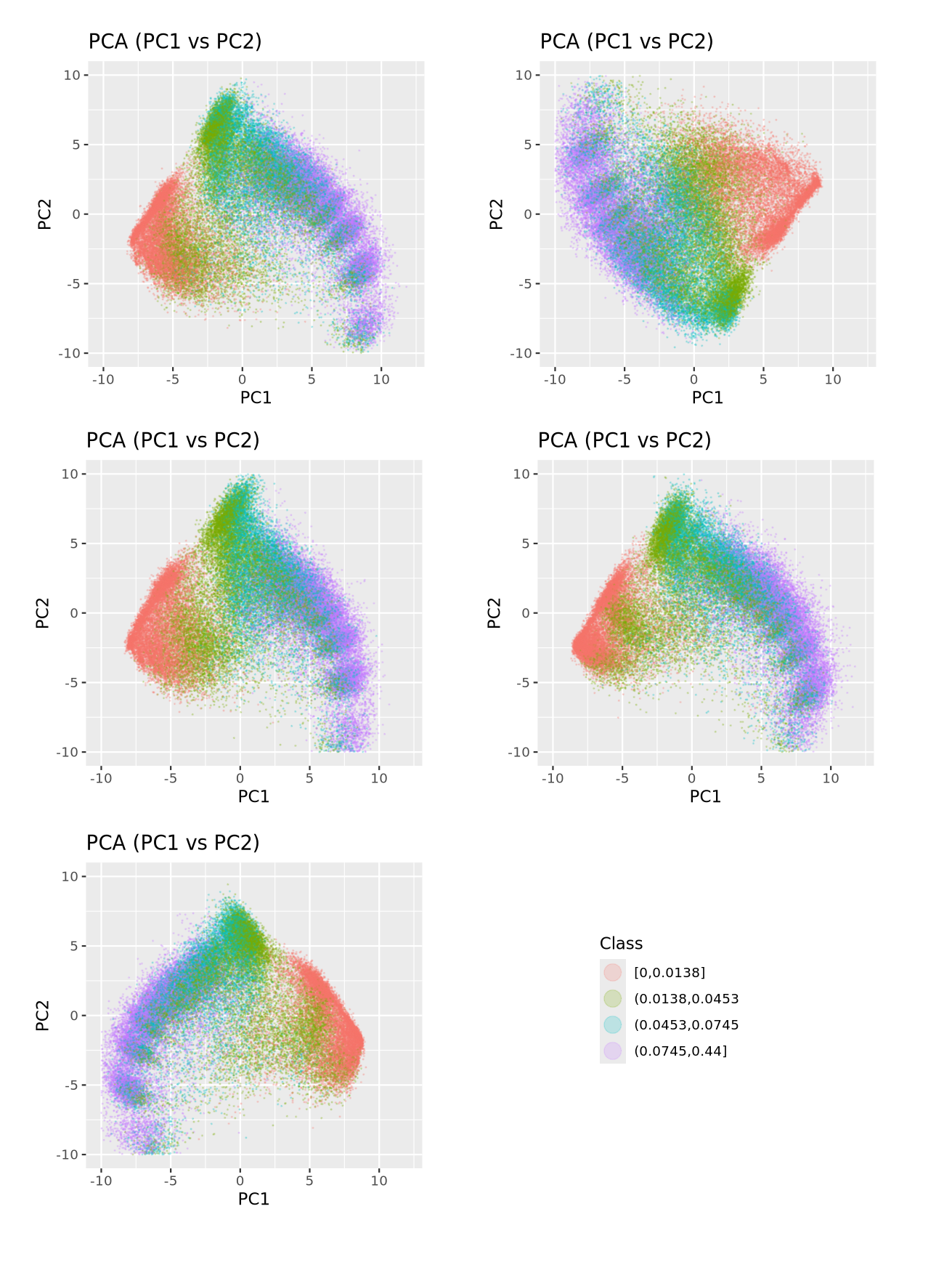
**

**Supp Figure 5** Principal Component Analysis (PCA) based clustering of all case embeddings from 5-fold models. Points colors were dimmed to highlight the cluster centers.


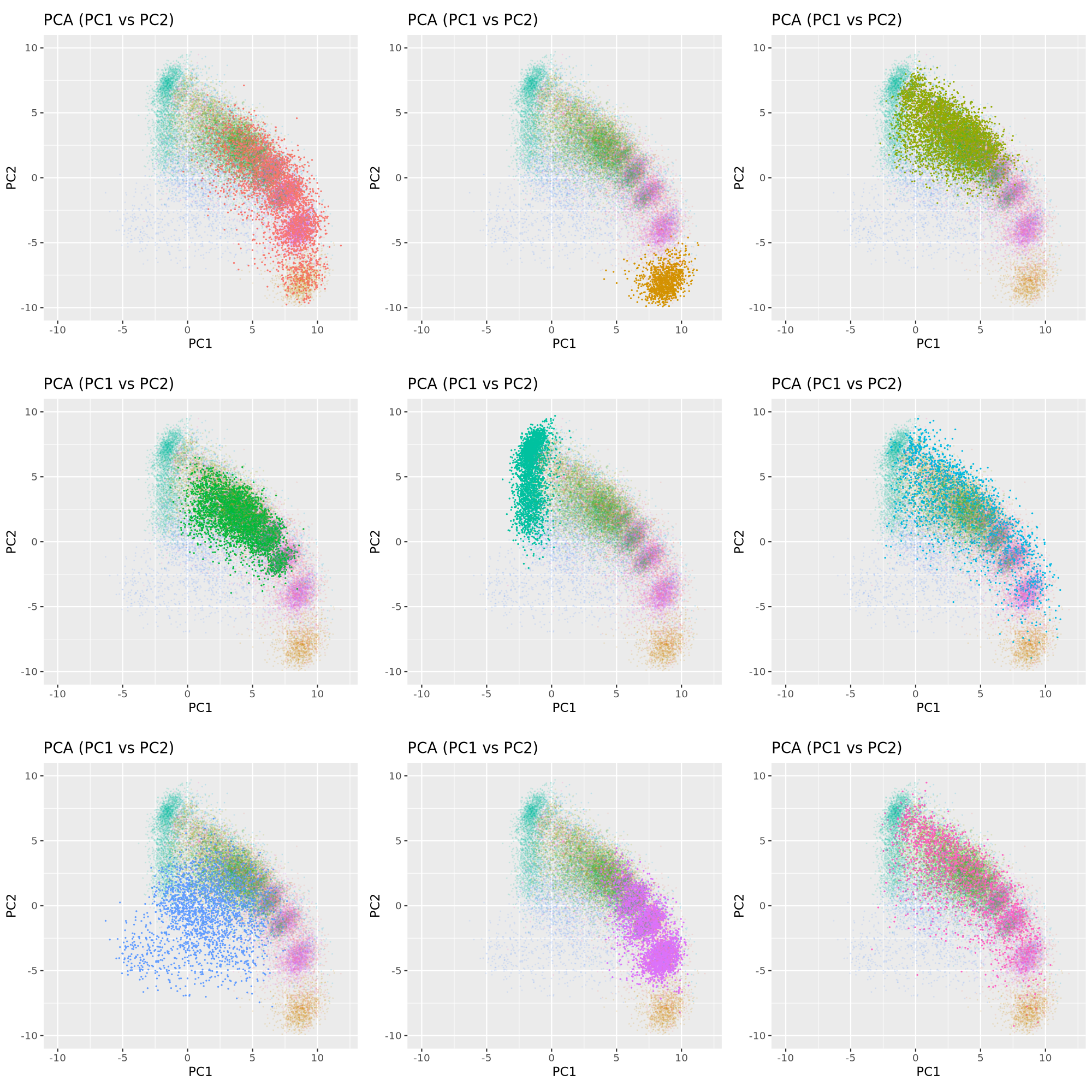


**Supp Figure 6** Gaussian Mixture Model (GMM) identified nine clusters of high quality pre-Anti-Obesity Medication (pre-AOM) periods, highlighted in separate plots on the two major PCs’ space.


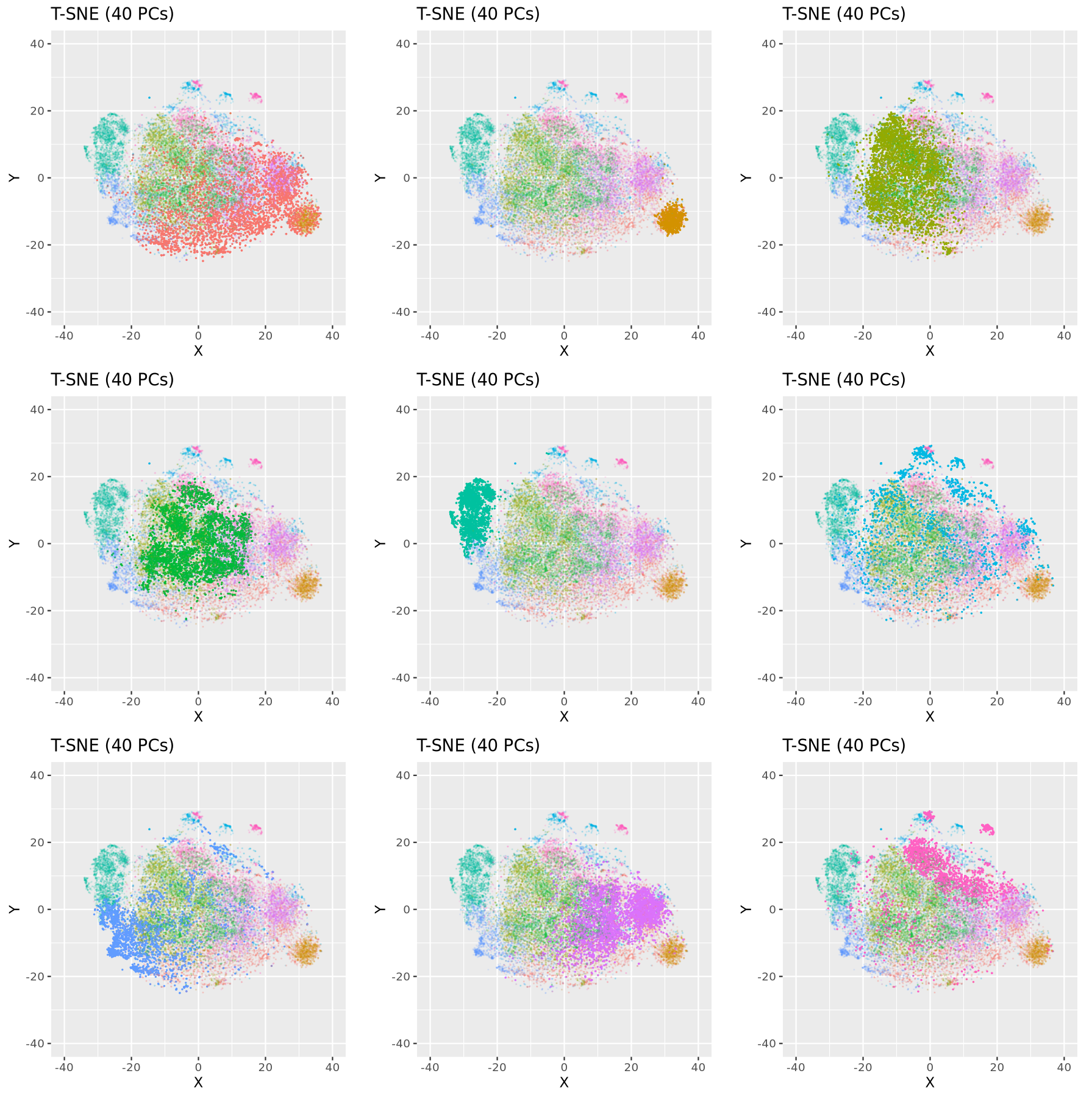


**Supp Figure 7** Gaussian Mixture Model (GMM) identified nine clusters (filled by row) of high quality pre-Anti-Obesity Medication (pre-AOM) periods, highlighted in separate plots on T-SNE space.

**
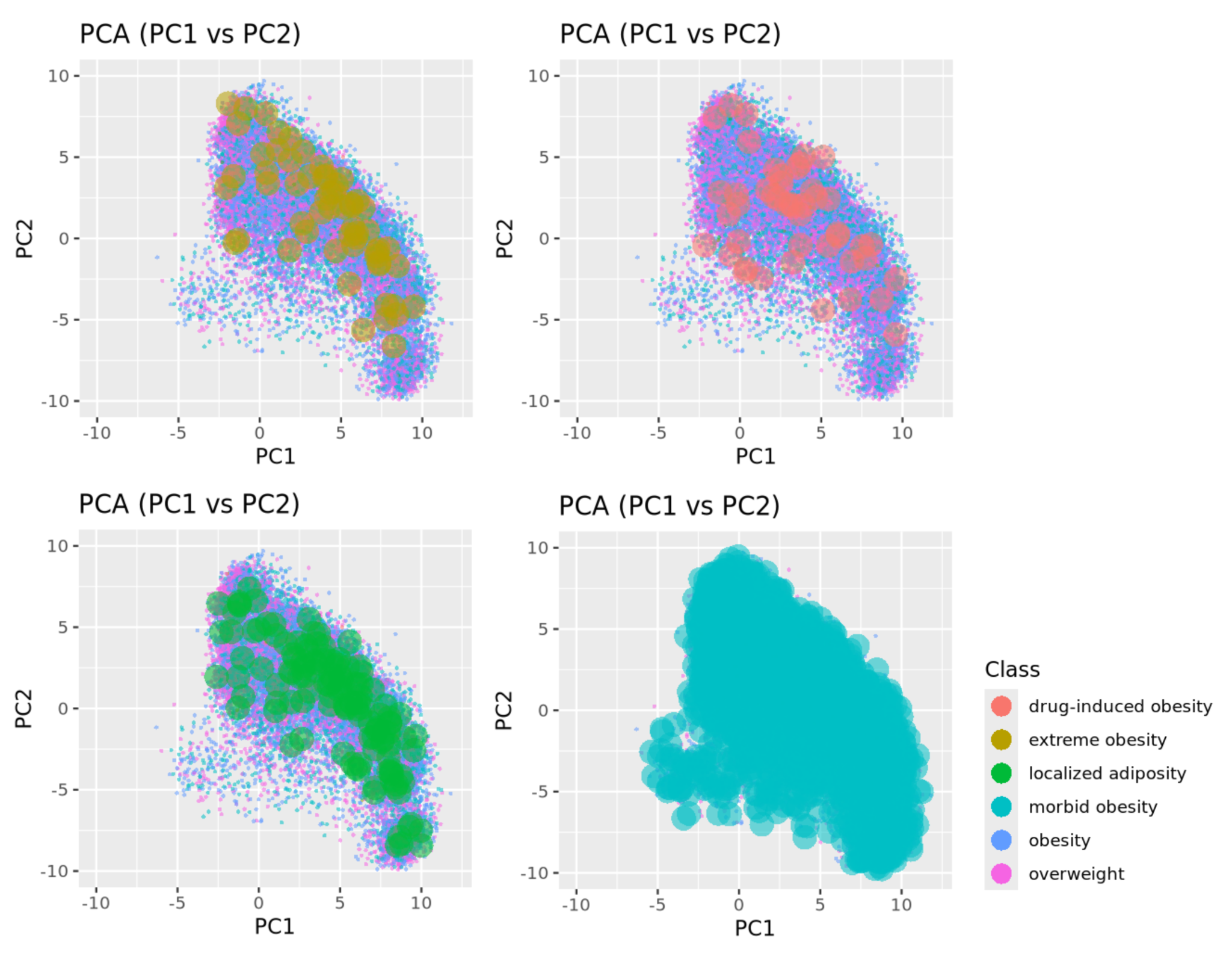
**

**Supp Figure 8** Distribution of traditional obesity phenotype in the context of GRU-D Autoencoder (GRU-D-AE) based clustering of pre-Anti-Obesity Medication (pre-AOM) periods.


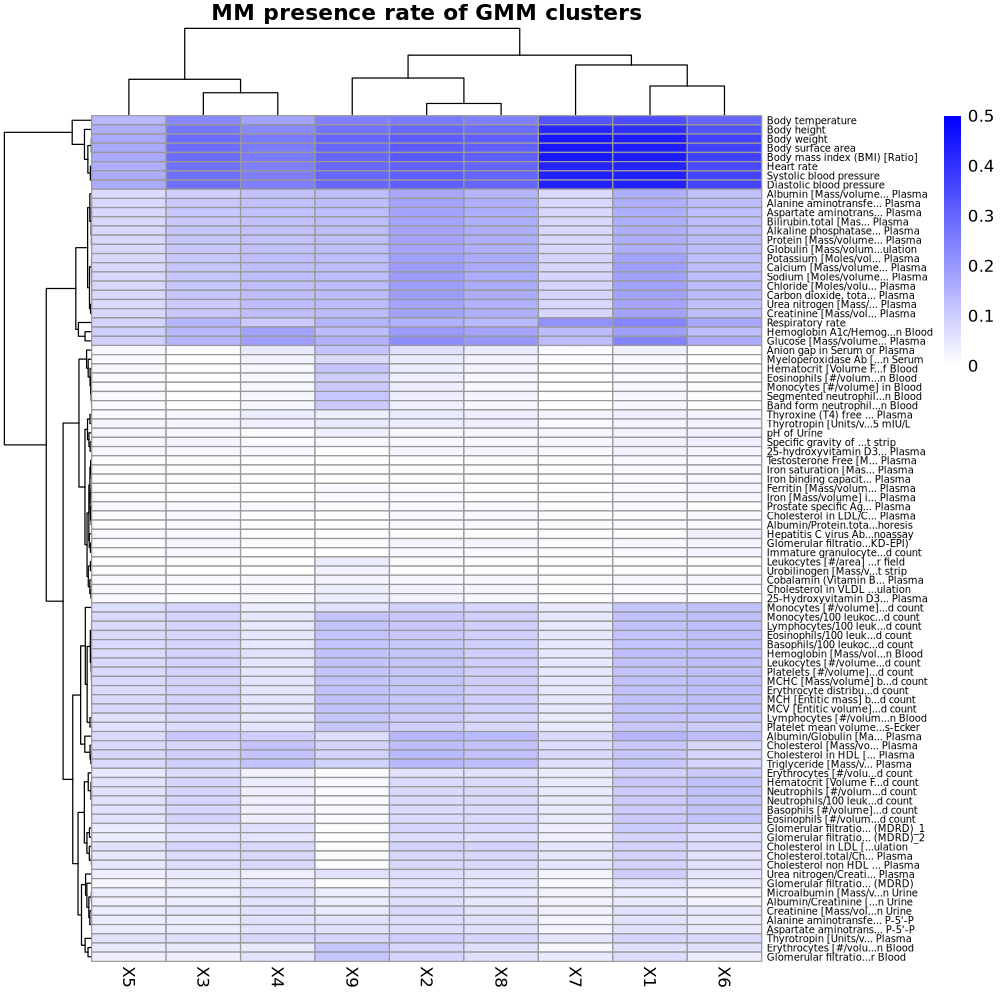


**Supp Figure 9** Average measurement presence rates against Gaussian Mixture Model (GMM) based clusters of pre-Anti-Obesity Medication (pre-AOM) periods. Presence rate is the proportion of measurements observed in the 13 time steps within 1 year before AOM initiation.

.


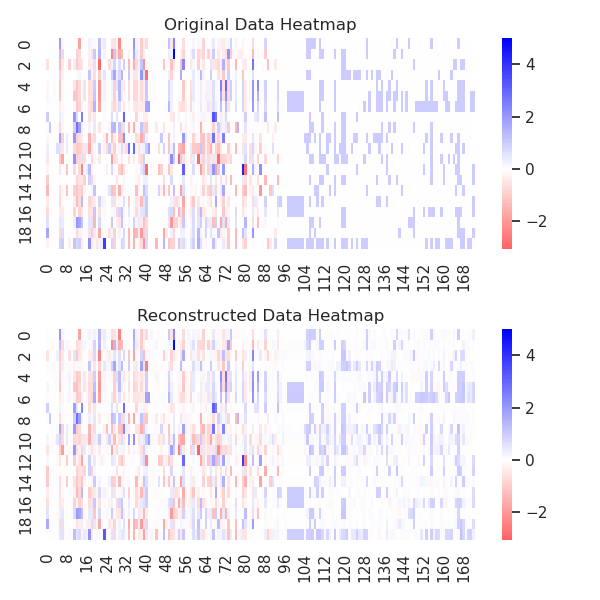


**Supp Figure 11** Static Autoencoder (SAE) based reconstruction of the static transformed Electronic Health Record (EHR) profiles during the pre-Anti-Obesity Medication (pre-AOM) period for 20 patients (y-axis) across 171 features (x-axis). Result displayed for one of the 5-fold models, with other folds performing similarly.

a b


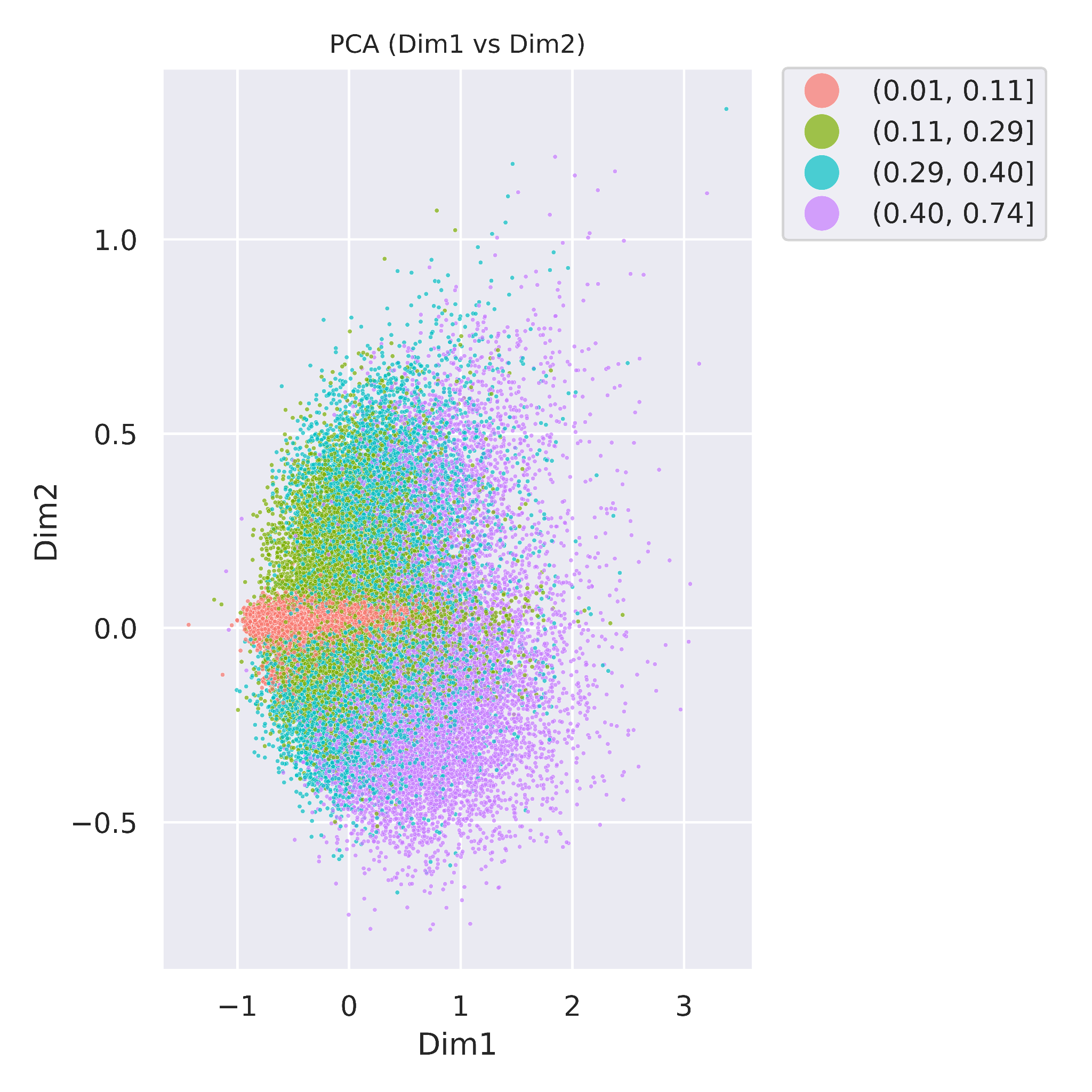

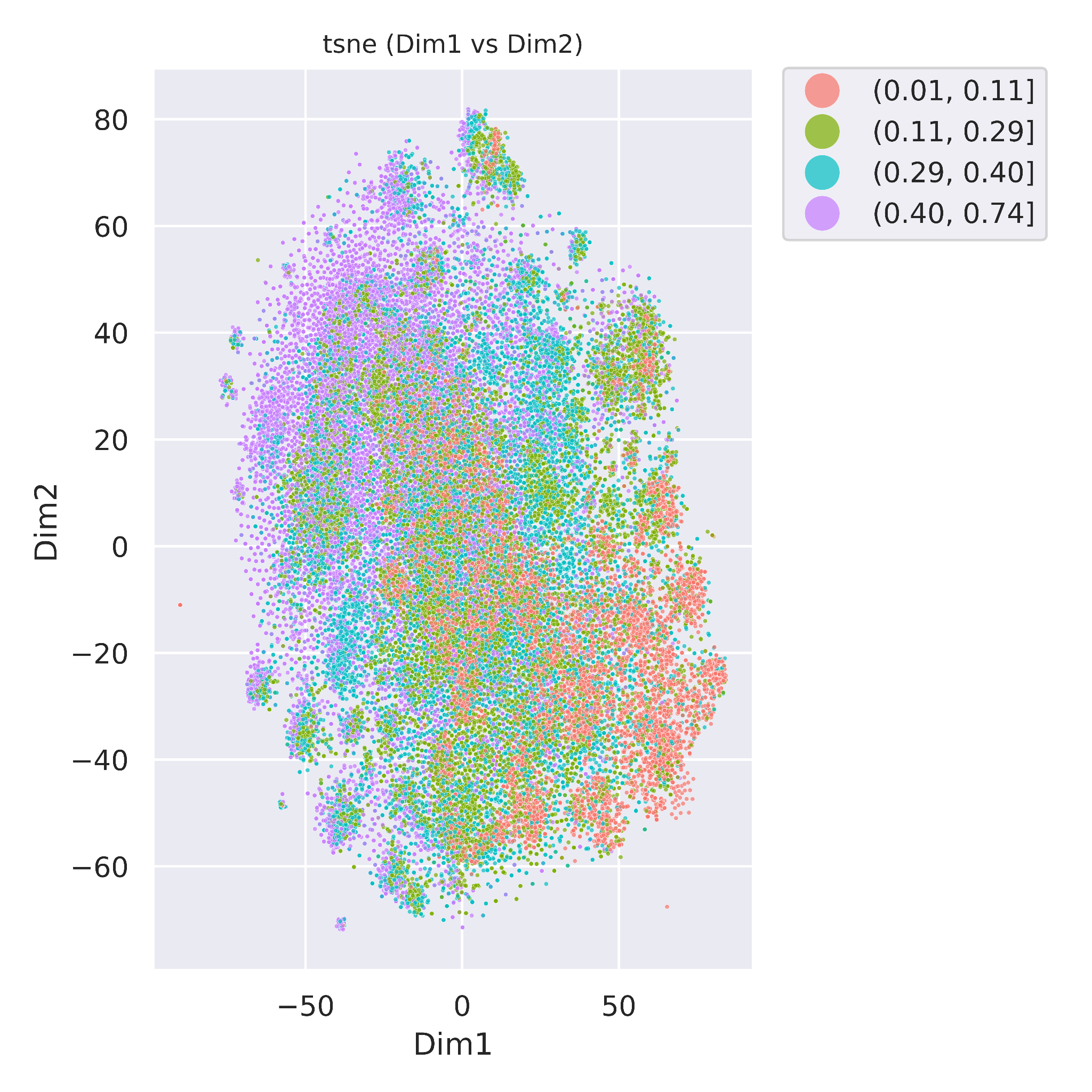


**Supp Figure 12** Static Autoencoder (SAE) based clustering of case pre-Anti-Obesity Medication (pre-AOM) periods colored by data quality quartiles. a) Top two Principal Components (PCs), b) T-SNE plot. Result displayed for one of the 5-fold models, with other folds performing similarly.

a b


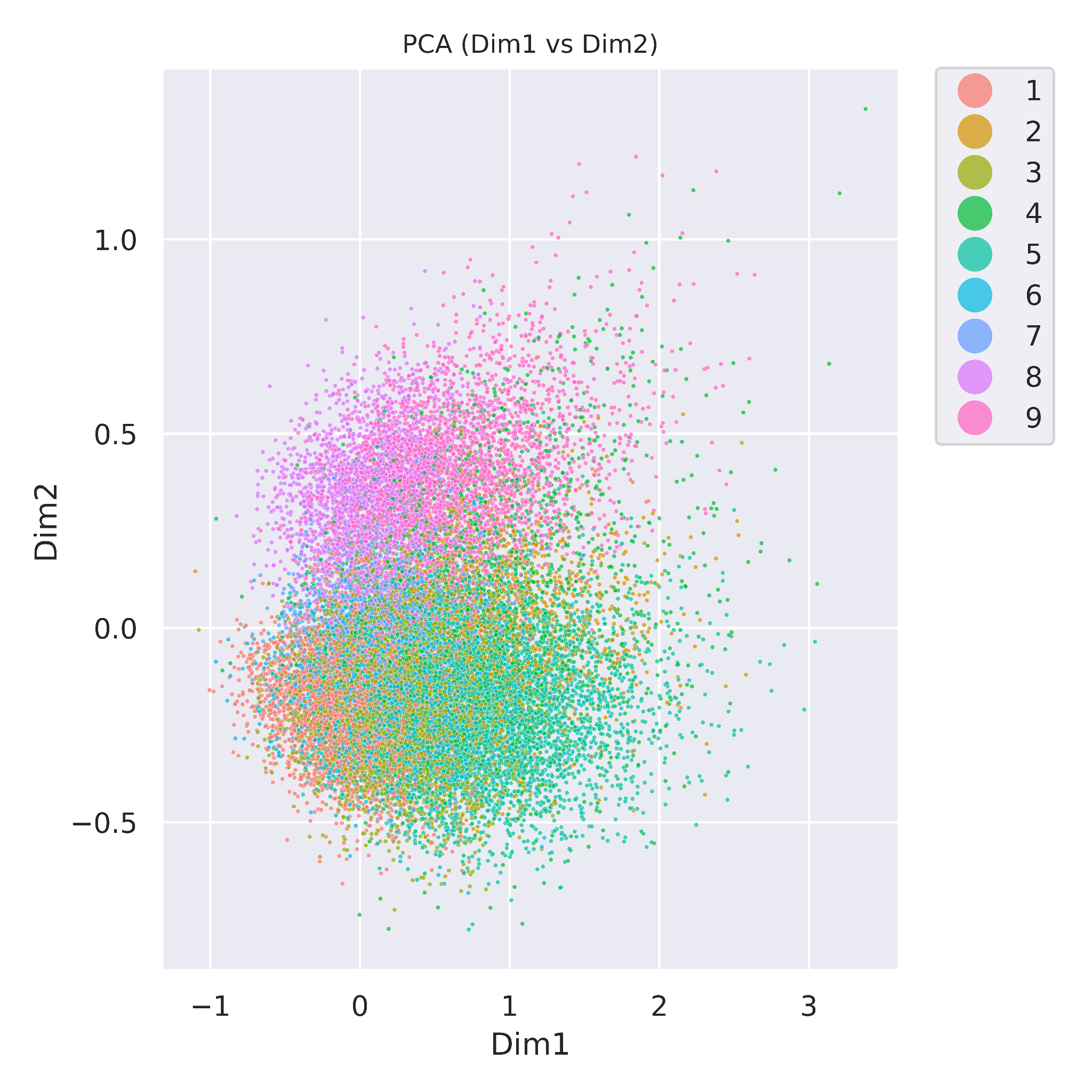

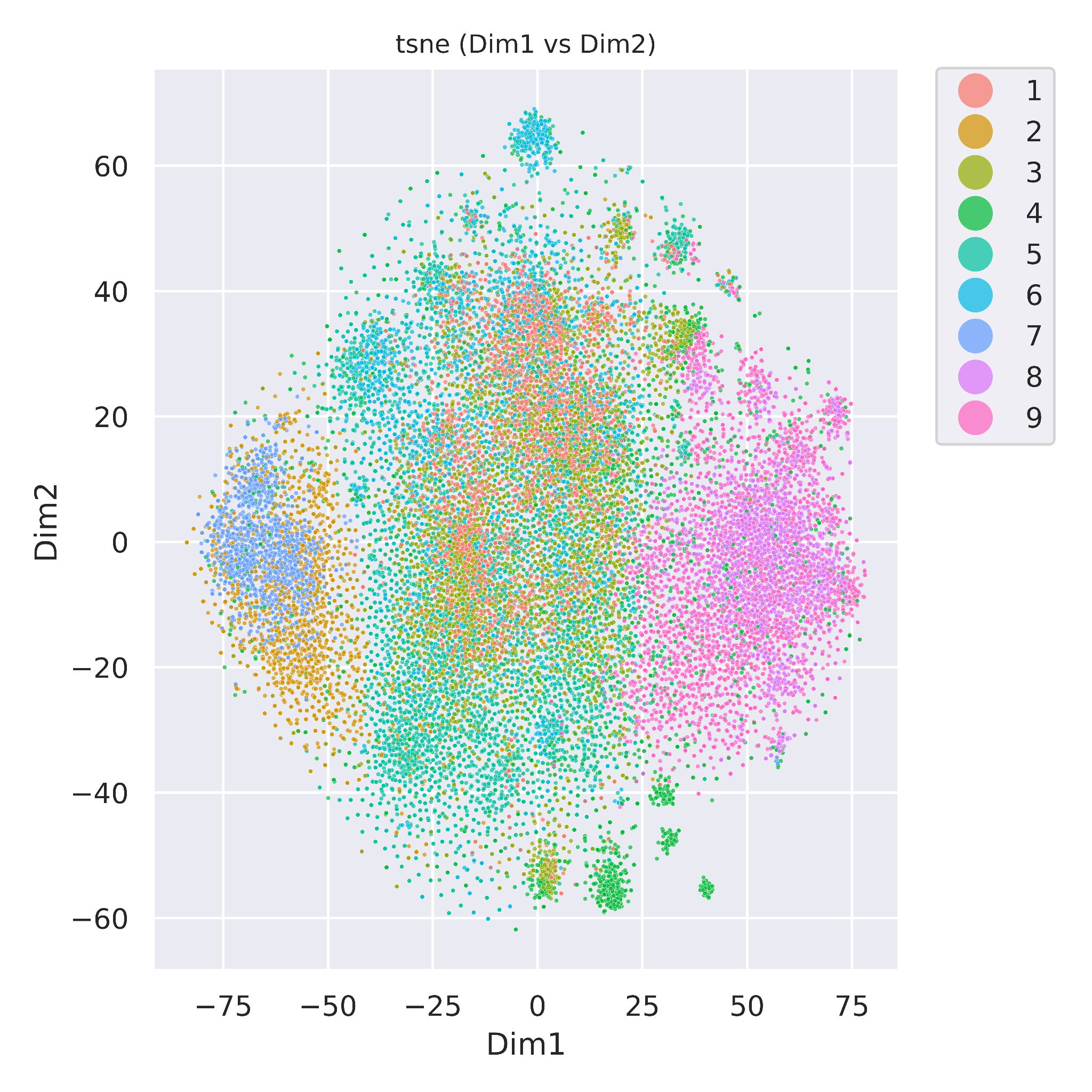


**Supp Figure 13** Static Autoencoder (SAE) based clustering of high quality cases pre-Anti-Obesity Medication (pre-AOM) periods colored by Gaussian Mixture Model (GMM) based clusters. a) Top two Principal Components (PCs), b) T-SNE plot. Result displayed for one of the 5-fold models, with other folds performing similarly.

a b


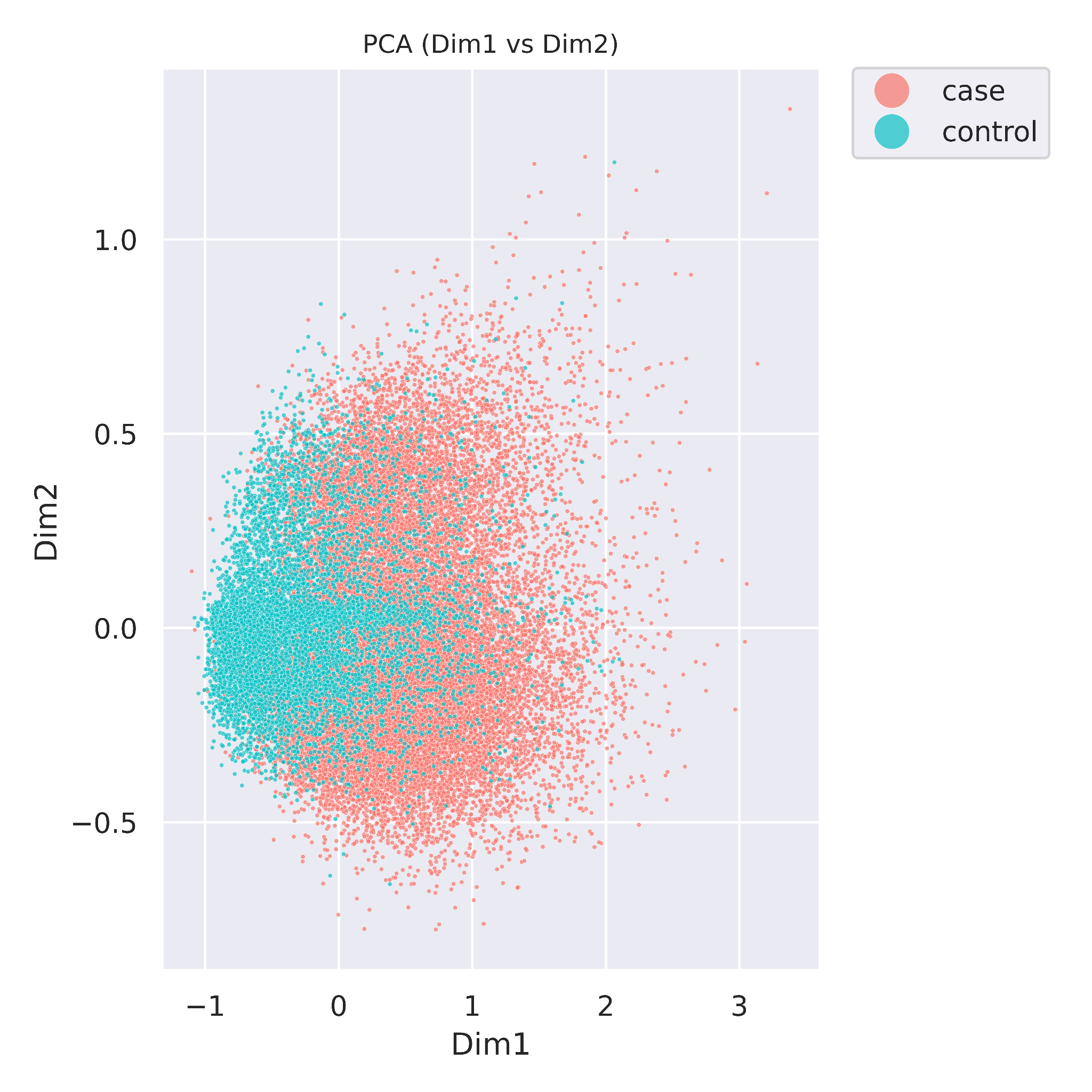

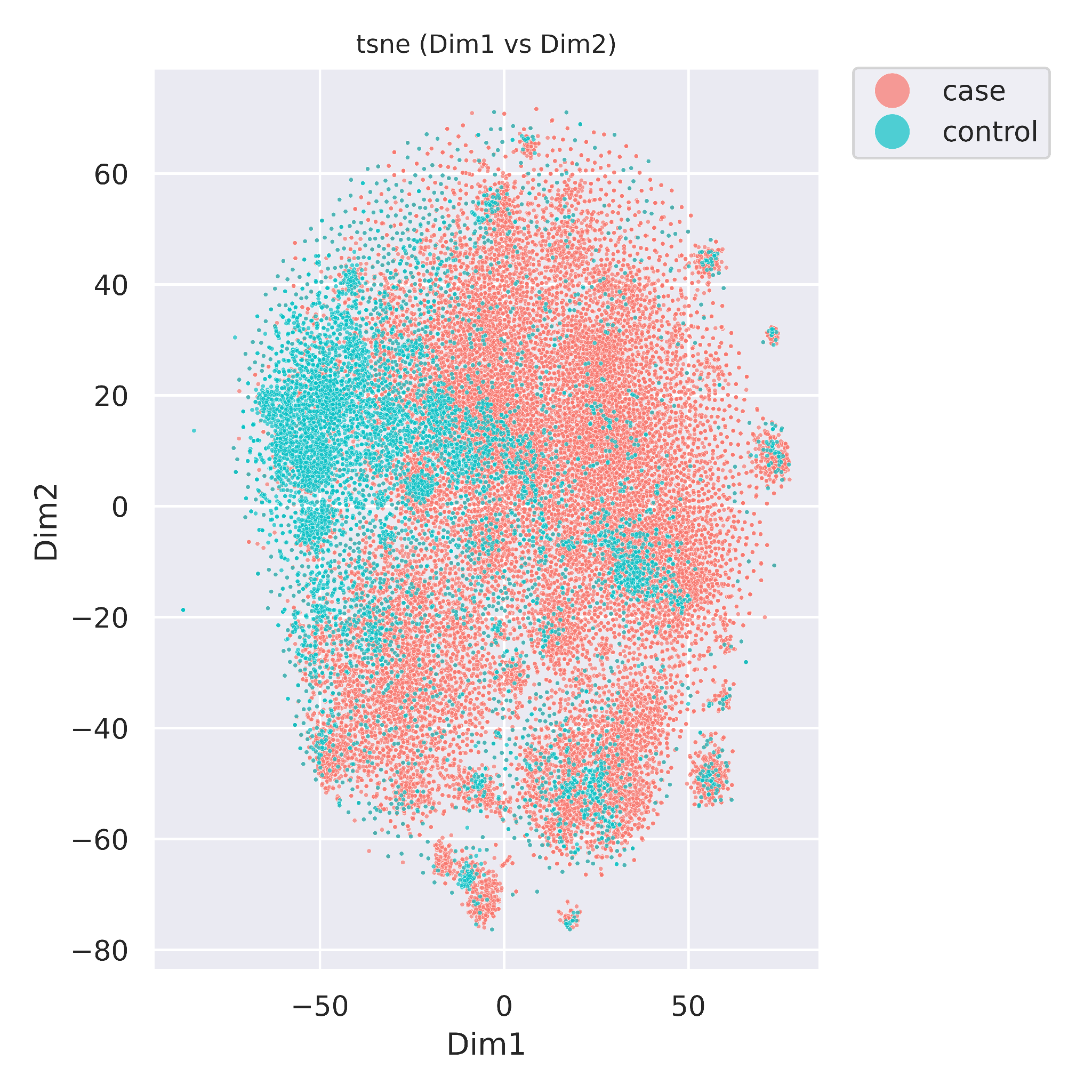


**Supp Figure 14** Static Autoencoder (SAE) based clustering of normal Body Mass Index (BMI) controls and high quality cases pre-Anti-Obesity Medication (pre-AOM) periods. a) Top two PCs, b) T-SNE plot. Result displayed for one of the 5-fold models, with other folds performing similarly.
